# A machine learning framework for discovery and enrichment of metagenomics metadata from open access publications

**DOI:** 10.1093/gigascience/giac077

**Published:** 2022-08-11

**Authors:** Maaly Nassar, Alexander B Rogers, Francesco Talo', Santiago Sanchez, Zunaira Shafique, Robert D Finn, Johanna McEntyre

**Affiliations:** European Molecular Biology Laboratory, European Bioinformatics Institute (EMBL-EBI), Wellcome Trust Genome Campus, Hinxton, Cambridge CB10 1SD, UK; Current affiliation: SciBite - an Elsevier Company, Wellcome Genome Campus, Hinxton, Cambridge CB10 1DR, UK; European Molecular Biology Laboratory, European Bioinformatics Institute (EMBL-EBI), Wellcome Trust Genome Campus, Hinxton, Cambridge CB10 1SD, UK; European Molecular Biology Laboratory, European Bioinformatics Institute (EMBL-EBI), Wellcome Trust Genome Campus, Hinxton, Cambridge CB10 1SD, UK; European Molecular Biology Laboratory, European Bioinformatics Institute (EMBL-EBI), Wellcome Trust Genome Campus, Hinxton, Cambridge CB10 1SD, UK; European Molecular Biology Laboratory, European Bioinformatics Institute (EMBL-EBI), Wellcome Trust Genome Campus, Hinxton, Cambridge CB10 1SD, UK; European Molecular Biology Laboratory, European Bioinformatics Institute (EMBL-EBI), Wellcome Trust Genome Campus, Hinxton, Cambridge CB10 1SD, UK; European Molecular Biology Laboratory, European Bioinformatics Institute (EMBL-EBI), Wellcome Trust Genome Campus, Hinxton, Cambridge CB10 1SD, UK

## Abstract

Metagenomics is a culture-independent method for studying the microbes inhabiting a particular environment. Comparing the composition of samples (functionally/taxonomically), either from a longitudinal study or cross-sectional studies, can provide clues into how the microbiota has adapted to the environment. However, a recurring challenge, especially when comparing results between independent studies, is that key metadata about the sample and molecular methods used to extract and sequence the genetic material are often missing from sequence records, making it difficult to account for confounding factors. Nevertheless, these missing metadata may be found in the narrative of publications describing the research. Here, we describe a machine learning framework that automatically extracts essential metadata for a wide range of metagenomics studies from the literature contained in Europe PMC. This framework has enabled the extraction of metadata from 114,099 publications in Europe PMC, including 19,900 publications describing metagenomics studies in European Nucleotide Archive (ENA) and MGnify. Using this framework, a new metagenomics annotations pipeline was developed and integrated into Europe PMC to regularly enrich up-to-date ENA and MGnify metagenomics studies with metadata extracted from research articles. These metadata are now available for researchers to explore and retrieve in the MGnify and Europe PMC websites, as well as Europe PMC annotations API.

## Introduction

Whole-genome shotgun DNA sequencing has emerged as a powerful tool for assessing the microbial content in a wide range of environmental samples. The advent of metagenomics analyses has enabled the phylogenetic and functional profiling of microbial communities found in a particular biome without the need of culturing. Meta-analysis between samples can reveal specific adaptations to environmental conditions or candidate members of the community that may be responsible for health or disease [1–3]. However, to establish causal relationships between observed phenotypes and compositional changes, potential confounding factors such as individual alcohol consumption or therapeutic interventions need to be considered [4,5]. Thus, to undertake meaningful analyses spanning multiple metagenomics data sets, accurate contextual metadata about the environmental conditions from which the sample was taken and the experimental methods are required [6]. Despite the efforts to deposit, organize, and analyze metagenomics data in public databases [7, 8], major obstacles to their usability thereof remain, due to incomplete, missing, and/or inaccurate contextual metadata.

Sequence databases, such as Sequence Read Archive [9] and European Nucleotide Archive (ENA) [10], provide permanent storage for metagenome sequences, which offer the potential for knowledge databases, such as MGnify [11], MG-RAST [12], and Integrated Microbial Genomes with Microbiome Samples [13], to explore the taxonomic diversity and metabolic pathways of biome microbial communities. Although significant advances have been made in the computation and indexing of metagenomics data, the lack of detailed and structured microbiome metadata has dramatically hampered metagenomics cross-study comparisons. To address this issue, metagenomics databases, such as Terrestrial Metagenome DB [14], Human Microbiome Project [15], Human Metagenome DB [16], Genomes OnLine Database [17] (GOLD), and MGnify, have used controlled vocabularies from ontologies, such as ENVO (Environmental ontology) [18], to manually annotate a broad range of metagenomics samples. However, many of these studies have incomplete detailed metadata about sample origins, due to the lack of metadata provided when depositing sequences into a database [19] and the inflexibility of hierarchical ontology relationships for describing diverse and specific environments; for example, ENVO lacks vocabularies that could describe samples from many engineered environments. A scalable way to overcome these shortcomings is the development of automated methods to extract sample and experiment metadata from research articles describing metagenomics studies and link them to metagenomics data sets.

Named Entity Recognition (NER) is one of the foundational text-mining methods used for retrieving information from the literature. In life sciences, traditional NER approaches have relied on annotating vocabularies onto texts [20, 21]. However, because of the limitation of their vocabularies, out-of-dictionary synonyms, and restrictive hierarchical levels, current ontologies fail to capture essential data pertinent to diverse metagenomic studies and hence the urgent need for machine-guided approaches [22]. Consequently, hybrid machine and deep learning approaches, such as Bidirectional Long Short-Term Memory (BiLSTM) and Conditional Random Field (CRF) models, have been recently recognized to efficiently identify diverse entities in biomedical texts. Principally, these NER classifiers have relied on generating context-independent word representations (embeddings) from unlabeled literature corpora using word2vec language models [23, 24], followed by the fine-tuning of these embeddings with hand-labeled data and task-specific neural architectures, such as LSTM [25, 26] or BiLSTM-CRF NER models [27–29]. But, whereas word2vec models aim to effectively capture the syntactic and semantic representations of words across diverse linguistic contexts, both context-independent and context-sensitive unidirectional representations [30] were found to be suboptimal for sentence- and token-classification tasks, where it is crucial to incorporate contexts from left and right directions (bidirectional representations) [31]—hence the emergence of the Bidirectional Encoder Representations from Transformers (BERT)–based models [32]. BERT alleviates both context-independent and context-sensitive unidirectional constraints with its unified architecture that can both pretrain and fine-tune bidirectional context-sensitive word representations for diverse Natural Language Processing tasks. BERT-pretrained word representations from English Wikipedia and Books Corpus managed to outperform hybrid architectures—context-independent or context-sensitive pretraining and BiLSTM-CRF fine-tuning models—in sentence- and word-classification (NER) tasks. Recently, BioBERT, a BERT-based model pretrained on PubMed abstracts and PMC full-text articles, has significantly outperformed BiLSTM-CRF and the original BERT models on most of the biomedical NER tasks [33]. Moreover, BioBERT has been recognized for its high performance in classifying medical entities after being fine-tuned with weakly supervised ontology-driven data [34].

Herein, we describe a machine learning framework capable of enriching sample and experimental metadata of a wide range of metagenomics studies with terms extracted from open access publications in Europe PMC [35]. This framework (i) classified and triaged publications describing research on diverse environments using machine learning models (literature classification), (ii) constructed manually curated metagenomics training sets for 16 novel metagenomics entity types, (iii) trained and validated BioBERT models to identify entities pertinent to the 16 metagenomics entity types in publications (NER), and (iv) developed and integrated a fully automated metagenomics annotations pipeline that regularly enriches up-to-date metagenomics studies in MGnify and ENA and provides researchers with model-predicted metadata from research articles in Europe PMC (databases enrichments).

## Materials and Methods

### Literature classification

#### Training data set

Due to the lack of public data sets for training metagenomics-specific literature classifiers, we constructed our own training data set based on MGnify, which provides access to a broad range of metagenomics studies and associated analysis results, but importantly curates microbiome studies with standardized biome annotations and provides direct links to their relevant publications. Accordingly, a supervised training data set was constructed by mapping the curated GOLD biome annotations assigned to the metagenomics studies in MGnify [36] to the corresponding publications in Europe PMC describing these studies (674 articles accessed on 17 February 2020). First, metagenomics studies were collected from MGnify with both PMC identifiers and GOLD annotations. Then, the full-text XML of publications corresponding to the PMC identifiers was retrieved with corresponding metadata from Europe PMC. Following XML preprocessing, sentences were parsed from the XML tags corresponding to each article section (i.e., introduction, method, results, discussion) and merged into individual section texts. Each text was aligned to the GOLD annotation of the corresponding metagenomic study, and TF-IDF (Term Frequency–Inverse Document Frequency) or a vector of precomputed 200-dimensional Doc2Vec embedding [37] was used as features for biome classifiers. To generate Doc2Vec embeddings, publications cited in MGnify records were trained using the document-embedding neural network model Doc2Vec with the following parameters: 15 training iterations, a window size of 5, worker threads of 8, and a dimensional size of 200 (Python genism package 3.8: Doc2Vec).

#### Training random forest biome classifiers on cross-referenced publications

A total of 5 GOLD multiclass hierarchical levels (Supplementary Table S1) were used to train nearly 50 random forest models (Python Scikit-learn package 0.22.1: RandomForestClassifier). For each of the TF-IDF and Doc2Vec approaches, a separate multiclass model was trained using the selected features for every hierarchical level. Each model trained a random selection of balanced data sets that comprised 20–100 texts per class. To validate model performance, datasets were divided into training data sets (80%) and test data sets (20%). The hyperparameter settings of random forest models were determined using a grid search among {50, 100, 150, 200, 250, 300} decision trees in the forest (n_estimators) and {25, 50, 75, 100, 125, 150, None} maximum tree depths (max_depth). Features selection was assessed through 5-fold shuffle-split cross-validation stratified by classes labels, and random state was fixed at model initialization for reproducibility. The precision (P), recall (R), and F1-score (F1) model performance metrics were compared and evaluated per hierarchical level.

#### Biome classifiers prediction and implementation

A query was constructed to retrieve all publications associated with all metagenomics studies from the ENA database (determined by the descriptor “library source: METAGENOMIC” via ENA browser [38] and ENA Cross Reference Service APIs [39]). This resulted in 4,513 publications from 9,949 ENA metagenomics studies accessed on 20 February 2020. For each publication, full texts were then retrieved and processed using the same workflow employed in constructing the classifiers training data set. The highest performing model with the broadest biome coverage was then selected to classify ENA cross-referenced publications. Using the model prediction probabilities, publications with probabilities of *P* ≥ 0.4 were selected to have higher publications coverage per category with 70–95% precision. Lastly, a random sample of ENA cross-referenced publications that represents the breadth of biome classes (44–50 publications per class) was manually verified to validate the predicted biomes and subsequently used for the literature triage (Supplementary Table S2).

### Named Entity Recognition (NER)

#### Training data set

A literature triage comprising 140 ENA cross-referenced publications was categorized into environmental (*n* = 50), engineered (*n* = 44), and host-associated (*n* = 46) publications (Supplementary Table S2). A total of 16 novel metagenomics entities, covering biome and experimental data, were proposed and defined for biocuration (Table 1). Words pertinent to the aforementioned entities were curated in the publications validated for biocuration by a team of experienced curators, using Hypothes.is [40]—a web-based open-source annotation tool. Every manuscript was assigned to a single curator to annotate the entities under the supervision of another curator, followed by revisions from 2 different curators (see supplementary curation guidelines). After evaluation, annotations were retrieved via the Hypothes.is API [41] and mapped to their corresponding sentences. Those sentences were then tokenized with BERT WordPiece tokenizer [42], which was modified to retain words that are unknown to the BERT model (e.g., long primers, kits, or chemical states with unknown characters), to be represented and trained by NER models instead of being excluded as unknown tokens (UNK). As entities can be nested within each other (overlapping entities), 16 individual training data sets were constructed to train each entity, separately. Each training data set consisted of all curated sentences with their entity-related tokens tagged, by the standard NER classification scheme BIO [43], as the beginning (B), inside (I), or outside (O) of an entity.

**Table 1:** Metagenomics: entity types

Entity	Definition
Ecoregion	Microbiome natural environment
Host	Microbiome living organism or host
Engineered	Microbiome humanmade environment
Date	Microbiome sample collection date
Place	The place of microbiome environment or host
Site	The site of microbiome sample within place
Body-site	The organ or tissue of microbiome sample
Sample-material	The material of the microbiome sample (e.g., water, mucus, soil)
State	The state of the microbiome environment or host (e.g., disease)
Treatment	Any treatment performed on the host (e.g., medicine) or the environment (e.g., fertilizer) from which the sample was collected
Kit	DNA extraction kit
Primer	PCR primers
Gene	Microbiome target genes (e.g., ribosomal RNA subunit and amplified region(s))
LS	Library source or library strategy (e.g., amplicon, whole genome)
LCM	Library construction method or layout (e.g., paired end, single end)
Sequencing	Sequencing platform

#### Training NER models on curated data sets

A total of 16 BioBERT models were trained to recognize metagenomics entities in publications, yielding 16 new metagenomics NER models. BioBERT pretrained weights generated from pretraining BERT on PubMed abstracts, and PMC full texts were fine-tuned with each of the 16 training data sets. To validate the performance of each model, each data set was divided into a training data set (90%) and test data set (10%). Fine-tuning was performed with a batch size of 32, maximum sequence length of 128, and fixed random state for reproducibility. A grid search was performed over 5 learning rates {1e-5, 2e-5, 3e-5, 4e-5} and 7 epochs {10, 30, 50, 70, 90, 110, 130} to select the highest performance hyperparameters for each model and thus different hyperparameters for each training model. The token-wise macro-average precision (P), recall (R), and F1-score (F1) were compared and evaluated per each learning rate and epoch combination, separately.

#### NER model prediction and implementation

**Figure 1:**
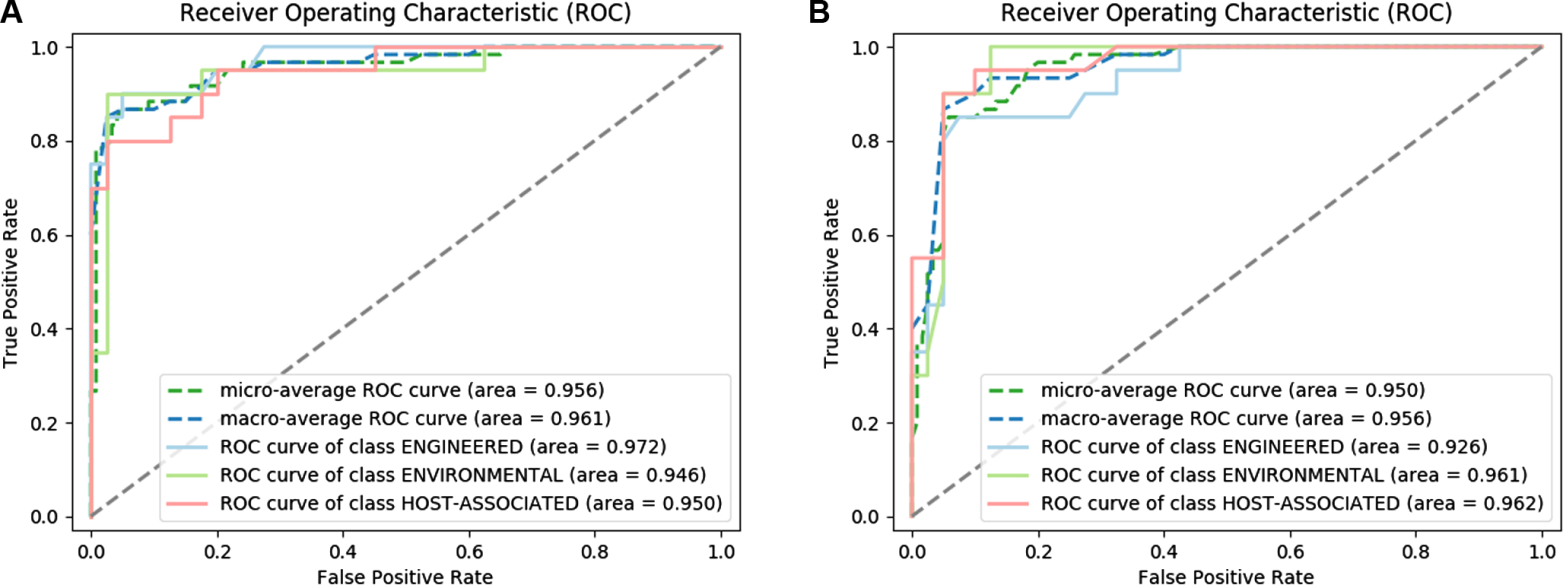
Receiver operating characteristic (ROC) curves of biome classifiers, using TF-IDF (A) or Doc2Vec^MGnify^ (B) as training features.

A total of 16 high-performance NER models (BioModels [44] accessions: MODEL2202160002, MODEL2202170001-MODEL2202170015) were used to annotate the methods sections of 114,099 metagenomics publications. Since the best model performance metrics were observed when biome classifiers were trained on method texts (see Supplementary Table S3), method sections were identified as the most representative article section for metagenomics data. The 114,099 metagenomics publications were identified as follows: (i) cross-referencing metagenomics studies in genome and biosamples databases (ENA, NCBI Taxonomy, BioProject, and MGnify) against literature repositories and (ii) querying literature repositories (Europe PMC, PMC, and PubMed) using metagenomics- and microbiome-related search and MeSH terms (for queries, see Supplementary Table S4). Then, methods sections were extracted from full-text XML via parsing the sentences wrapped within methods XML tags. Because metagenomics publications sometimes lack scientific methods sections, a multiclass random forest model was trained to classify full text into abstract-, introduction-, methods-, results-, and discussion-related texts. This section classifier (see Supplementary Table S5 and Figure 1) was then used to extract methods-related text from publications lacking methods sections.

#### Training NER models on predicted data sets

To further demonstrate the accuracy of predicted annotations, a random subset of new metagenomics publications (1,500 articles) was retrieved with their NER model-predicted annotations to train new 16 NER BioBERT models from scratch. To construct this subset, trained biome classifiers were used to classify new metagenomics publications into 3 broad biome environments. Then, a random subset of 500 articles, with predicted probabilities of *P* ≥ 0.5, was selected per biome class (i.e., engineered, environmental, and host-associated classes), followed by the retrieval of their NER model-predicted annotations. In this approach, those new publications with their NER-predicted annotations were considered as the training data set, whereas the curated data set was considered the test data set. This approach is like the student self-distillation process used in improving ImageNet classification [45] and protein structure prediction (AlphaFold) [46] models, where student models were trained on data sets predicted by teacher models (models trained on the curated data set). In our approach, however, it was hypothesized that teacher NER models—models trained on curated data sets—could generalize to novel biome publications if student NER models—models trained on data sets predicted by teacher models—predicted curated data sets with token-wise macro-average F1-score ≥70%.

### Database enrichment

#### Europe PMC enrichment

Metagenomics annotations of 114,099 publications in Europe PMC were represented in the annotations data model provided by the Europe PMC annotations submission system [47]. To make it easier to categorize and search publications with metagenomics annotations, specific metagenomics ontologies were required to standardize the predicted annotations. However, with the surge in different environments being sampled, it became clear that metagenomics ontologies, including GOLD and ENVO, are insufficient for standardizing most of the predicted annotations. Thus, ZOOMA [48], a semantic ontology mapping tool, was used to map each of the predicted nonstandardized annotations to a wide range of ontologies in the Ontology Lookup Service [49]. Processed annotations were then uploaded into the Europe PMC annotations API for users to search, explore, and retrieve programmatically.

#### ENA and MGnify enrichment

Metagenomics annotations of publications describing 19,209 studies in ENA were amassed from the Europe PMC annotations API. To assess the enrichment of experimental and sample metadata, metagenomics annotations (NER model-predicted annotations) were compared with the corresponding author-submitted metadata for 19,209 metagenomics studies in ENA. However, no sample or experimental metadata were associated with nearly 2,810 studies in ENA. For each study, sample, experiment, and run records identifiers—accessions—were parsed from ENA-SAMPLE, ENA-EXPERIMENT, and ENA-RUN tags in the study XML, respectively, followed by the retrieval of run and experimental metadata from the identifier-associated records. Then, the entities of metagenomics annotations and a subset of ENA fields used in more than 50 studies were mapped to a corresponding subset of the minimum information about any (x) sequence (MIxS) [50] checklist (see Supplementary Table S7). For each MIxS term, data were pooled from mapped ENA fields (author-submitted metadata) and metagenomics entities (metagenomics annotations) and compared per study. Studies with any identical or nonidentical metadata in both sources or having metadata from one source only were then counted per each MIxS term, with the results from the 20 most common MIxS fields shown in Figure 4.

## Results

### Overview

A machine learning framework was developed and integrated for enriching a wide variety of metagenomics studies with essential and accurate metadata from open access research articles. This framework (i) trained machine learning models on cross-referenced data to classify a broad range of biome publications (literature classification and triage), (ii) defined and curated 16 novel bioentities that describe vital metadata for diverse metagenomics studies (biocuration; Table 1), (iii) trained and validated BioBERT-based NER models on curated data sets (BioBERT fine-tuning and internal validation), (iv) deployed 16 high-performance trained NER models in retrieving accurate metagenomics metadata from thousands of publications in Europe PMC (NER), (v) standardized BioBERT model-predicted annotations with multiple domain-specific ontologies (normalization), (vi) validated the transfer of model accuracy and reliability to uncurated data sets in Europe PMC and ENA (external validation), and (vii) developed an integrated and fully automated metagenomics annotations pipeline that regularly enriches up-to-date metagenomics studies (19,209) with model-predicted metadata from research articles (114,099) (databases enrichments).

### Literature classification

Predicting a wide variety of microbiome environments was an essential requirement to triage publications for NER tasks. This requirement was addressed by constructing supervised training data sets, where the GOLD annotations assigned to metagenomics studies were mapped to the corresponding MGnify cross-referenced publications. Subsequently, random forest models were trained on the hierarchical levels of GOLD ontology, yielding diverse biome prediction models. Comparing model performance metrics revealed higher F1-scores of 91–97% for the top GOLD hierarchical levels (Engineered, Environmental, and Host-associated). However, Doc2Vec models were outperformed by their TF-IDF alternatives (Table 2, Figure 1). Hence, TF-IDF models were applied for the prediction of microbiome environments in new metagenomics publications (selected based on the publication being referenced from an ENA study record) and subsequently literature triage.

**Table 2:** Precision, recall, and F1-scores of the best-performing random forest biome classifiers

Classifier features	Class	Precision	Recall	F1-score	Support
TF-IDF	Engineered	0.86	0.9	0.88*	20
	Environmental	0.86	0.9	0.88*	20
	Host-associated	0.94	0.85	0.89*	20
Doc2Vec^MGnify^	Engineered	0.77	0.85	0.81	20
	Environmental	0.89	0.8	0.84	20
	Host-associated	0.85	0.85	0.85	20

Classifier features were either TF-IDF or Doc2Vec^MGnify^ (embeddings generated from MGnify cross-referenced publications). Support = number of publications in test data sets per class. *TF-IDF biome classifiers outperformed the ones trained on Doc2Vec^MGnify^. n_estimators = 300 (TF-IDF) and 250 (Doc2Vec^MGnify^). max_depth = 25. Random state = 9.

### Named Entity Recognition (NER)

To identify metagenomics data in research articles, 16 novel metagenomics entities (Table 1) were defined, curated, and trained using NER models. Training and test data sets were constructed from the curated 140 ENA cross-referenced publications, which were not contained in the MGnify database to ensure unbiased and broader coverage of biome. Triaged publications (see Supplementary Table S2) were randomly selected and equally categorized using highest performance random forest classifiers (Table 2, Figure 1). The manual curation of metagenomics entities in literature triage yielded 9,567 annotations for 2,496 sentences. As entities can be nested within each other, 16 individual data sets were constructed separately for fine-tuning 16 BioBERT models. Each entity data set, comprising 2,496 BERT-tokenized sentences with BIO-tagged entities, was partitioned for training (90%; 2,246 sentences) and testing (10%; 250 sentences). During training, grid search over 5 learning rates and 7 epochs showed best performance models with diverse combinations of hyperparameters per entity. These varieties of combinations were expected to give the best performance, given the overlapping nature of the entities. Typically, the greater the contexts overlap, the greater the training the model required to achieve the best performance. Table 3 shows the best token-wise precision (P), recall (R), and F1-score (F1) macro-averages for trained NER models per entity. Models achieved a precision of 80–100% and an F1-score of 71–98%.

**Table 3:** Token-wise: precision, recall, and F1-score of the 16 best-performing NER models

Entity	Learning rate	Epoch	Recall	Precision	F1-score
Ecoregion	4e-5	50	0.95	1	0.98
Host	2e-5	90	0.89	0.93	0.9
Engineered	2e-5	10	0.65	0.93	0.75
Date	4e-5	90	0.78	0.91	0.83
Place	3e-5	90	0.78	0.86	0.82
Site	4e-5	10	0.71	0.85	0.77
Body-site	4e-5	90	0.98	0.95	0.97
Sample-material	5e-5	110	0.8	0.9	0.85
State	5e-5	110	0.65	0.8	0.71
Treatment	4e-5	30	0.66	0.8	0.73
Kit	2e-5	70	0.94	0.91	0.92
Primer	5e-5	70	0.94	0.97	0.96
Gene	1e-5	10	0.86	0.92	0.89
LS	5e-5	50	0.8	0.95	0.85
LCM	4e-5	50	0.86	1	0.92
Sequencing	5e-5	110	0.84	0.89	0.87

To validate the accuracy of NER models predictions, 16 new NER models were trained from scratch on uncurated predicted annotations from 1,500 new articles. The trained models were then tested in recognizing entities in the curated data sets (140 articles that were not included in the 1,500 training articles). Supplementary Table S6 shows the best token-wise P, R, and F1 macro-averages for the NER models trained on predicted entities, where F1-scores were 73–97%. These NER models outperformed those trained on the curated data sets for *date,place,state,treatment,kit,gene,Library Strategy (LS),Library Construction Method (LCM*), and *sequencing* entities.

### Database enrichment

To enrich metagenomics studies with data from research articles, 114,099 publications in Europe PMC were processed and annotated using the highest NER performance models (Table 3). Those publications encompassed 19,900 publications linked to studies in the ENA and MGnify databases.

**Figure 2:**
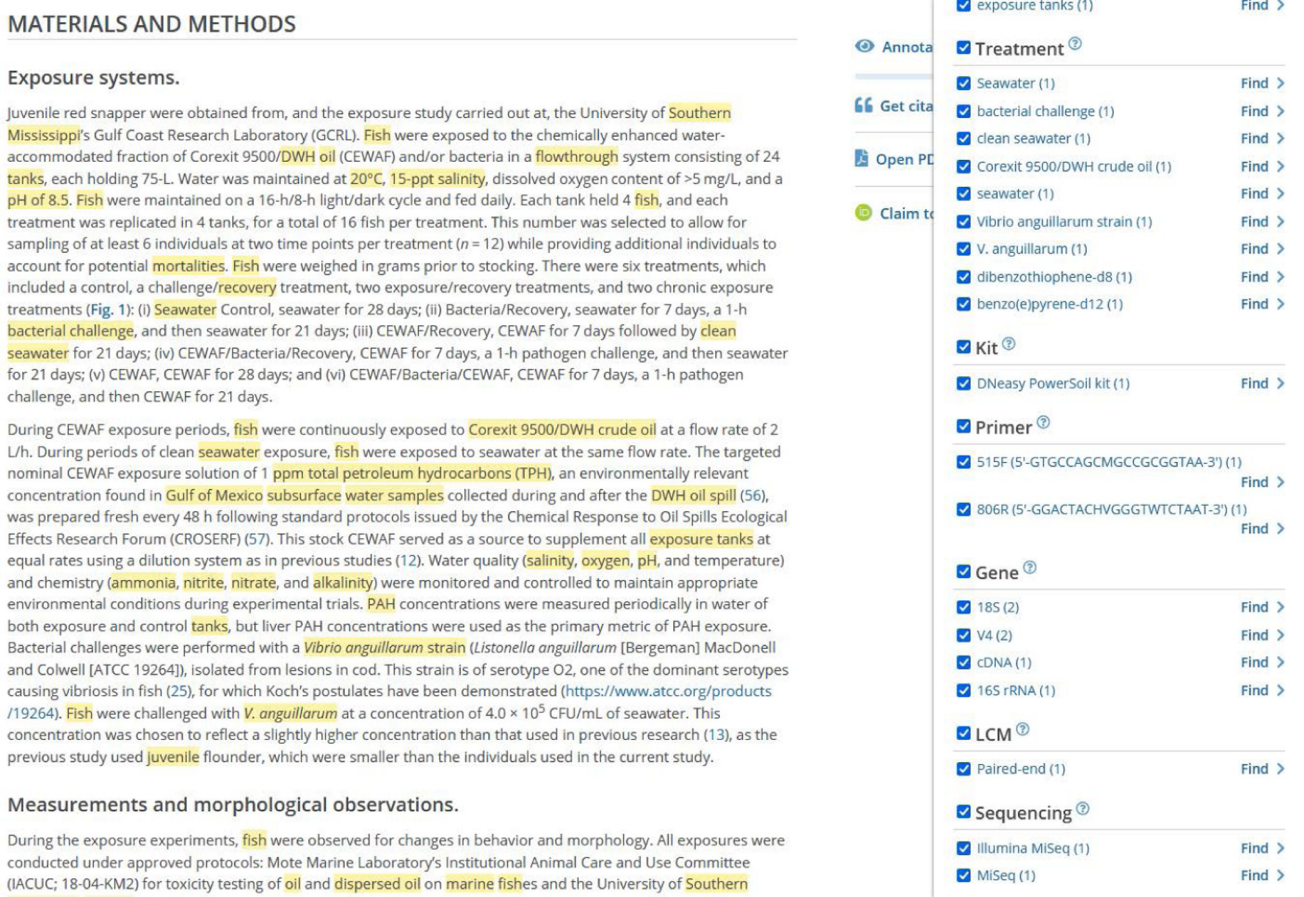
Screenshot of Europe PMC article. Annotations panel with metagenomics entities and annotations (right). Highlighted annotations in full-text using SciLite tool (left) (SciLite article view from PMC8791192).

A new pipeline was developed to continuously provide metagenomics annotations for new open access publications. Users are now allowed to search, explore, and retrieve metagenomics annotations programmatically from the Europe PMC website, using search queries (e.g., ANNOTATION_PROVIDER: “Metagenomics” [51]), a SciLite application (Figure 2: article view), and the annotations API [52], respectively. The pipeline was tested on a Linux operating system and Google Colab GPUs and CPUs. A minimum of Python 3.6 or 3.7 is required. In addition, if Conda will be used as a Python packages manager, then a minimum version of Miniconda 4.7.10 can be installed to create a Python 3.7 environment with all the necessary packages. A more detailed description about the necessary packages can be found under the “Availability of Source Code” section and the project GitLab repository.

**Figure 3:**
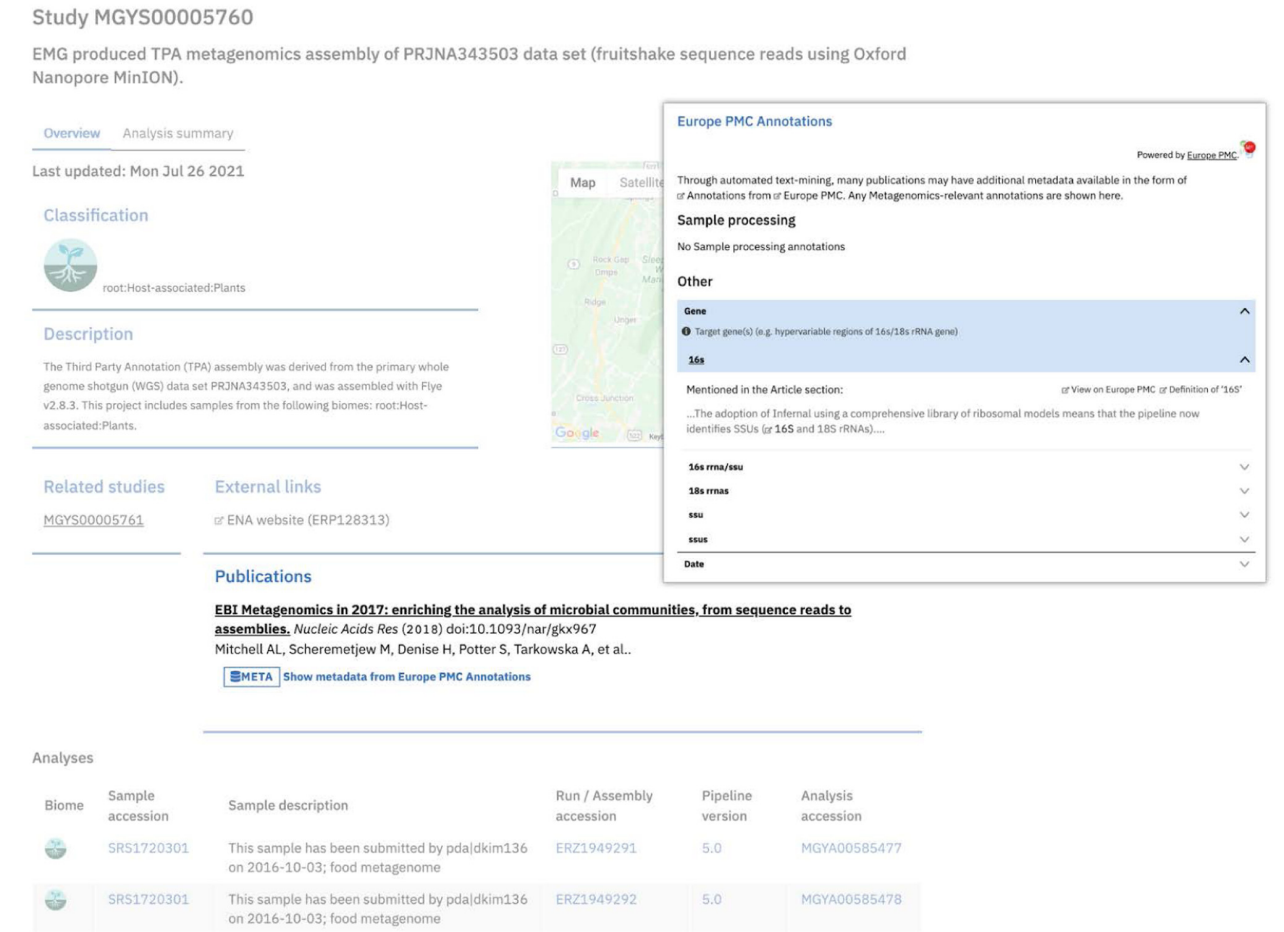
Composite screenshot showing annotations-enriched metadata for a MGnify study. Annotated publications are highlighted within a study (left) and annotations are shown in context (right).

To date, MGnify has enriched the metadata of 2,310 studies with the metagenomics annotations from corresponding articles (1,800) using the Europe PMC annotations API. Enriched metadata were made accessible in MGnify publications [53] and sample web pages (Figure 3: study view) for researchers to explore in metagenomics analyses. Moreover, a total of 1,658,023 of metagenomics annotations were linked to BioSamples records and deposited in ELIXIR Contextual Data Clearinghouse (under curations and provider Name = EMERALD) [54] for curators to validate and integrate into ENA.

**Figure 4:**
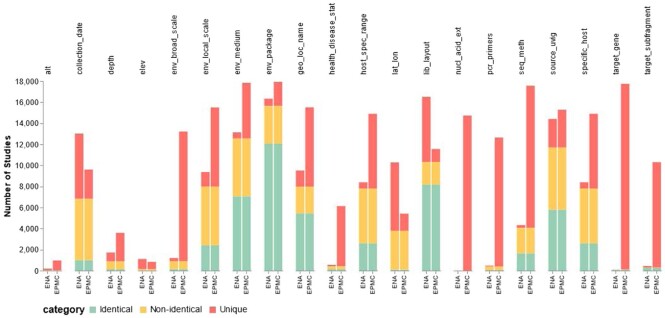
A clustered stacked bar plot showing the overlap of metadata terms between ENA (author submitted terms that are part of the study) and those derived from Europe PMC (EPMC) articles using our framework. For each study, we have evaluated 20 of the most populated MIxS fields in 19,209 studies, to establish if the metadata provided by both sources were identical (green), nonidentical (yellow), or provided by one of the metadata sources only (unique, red). For each MIxS field, each stacked bar shows the number of studies having identical, nonidentical, and unique metadata from each source.

To further evaluate our approach, the publication-derived metagenomics data were compared against author-submitted metadata for 19,209 studies. Both sets of metadata were mapped to the relevant MIxS checklist, allowing the annotations to be compared. Since both publication-derived metagenomics data and author-submitted metadata were provided as free text, comparing them was a nontrivial task and hence the comparison of exact matches. This comparison revealed that for the vast majority of MIxS checklist terms, the numbers of publication-derived terms were greater than the numbers retrieved from ENA author-submitted metadata (Figure 4; see Supplementary Figure S4 data, Figure 4 analysis data). In addition, metagenomics annotations demonstrated high-quality coverage for many of the missing metadata in ENA, such as polymerase chain reaction (PCR) primers (pcr_primers), phylogenetic marker genes (target_gene), nucleic acid extraction kit (nucl_acid_ext), environment phenomena (state), health or disease states (state), and treatment (Table 4). Moreover, overlaps were observed between metagenomics annotations and ENA metadata in numerous studies regarding MIxS env_package (12,062 studies), lib_layout (8,173 studies), env_medium (7,058 studies), source_uvig (5,789 studies), geo_loc_name (5,445 studies), specific_host (2,597 studies), host_spec_range (2,597 studies), env_local_scale (2,412 studies), seq_meth (1,649 studies), collection_date (1,002 studies), target_subfragment (241 studies), env_broad_scale (144 studies), health_disease_stat (126 studies), depth (120 studies), lat_lon (91 studies), target_gene (80 studies), pcr_primers (58 studies), elev (26 studies), alt (2 studies), and nucl_acid_ext (1 study). Yet, many studies have shown no or limited overlap, either due to missing essential metadata or having inaccurate, incorrect, or inconsistent metadata (i.e., synonyms or unit formats) in ENA compared with their metagenomics annotations counterparts (see Supplementary Table S8).

**Table 4:** Some examples of how the metagenomics annotations extracted from the publications describing ENA records enrich the metadata for those studies.

Study	PMCID	MIxS (entity type)	ENA metadata	Metagenomics annotations
PRJDB8863	PMC6941062	pcr_primers (primer)	–	RNA (ribosomal RNA) genes (forward: 5′-ACACTCTTTCCCTACACGACGCTCTTCCGATCTGTGCCAGCMGCCGCGGTAA-3′; reverse: 5′-GTGACTGGAGTTCAGACGTGTGCTCTTCCGATCTGGACTACHVGGGTWTCTAAT-3')
PRJDB9293	PMC8147061	target_gene (gene)	–	16S ribosomal RNA, V3–V4
PRJDB5614	PMC5745017	nucl_acid_ext (kit)	–	RNA PowerSoil total RNA isolation kit
PRJEB27411	PMC6072794	Env_package (state)	–	100-year drought, 75.6 mm of rainfall, mesotrophic, denitrification
PRJEB15392	PMC5405060	health_disease_stat (state)	–	Caries, gingivitis, medically healthy, oropharyngeal mucositis, poor oral hygiene, pulpal diseases
PRJEB22207	PMC7044117	Env_package (treatment)	–	Antibiotic, benzylpenicillin, cefotaxime, gentamicin, meropenem, metronidazole, probiotic supplementation, vancomycin
PRJDB10581	PMC8151423	env_local_scale (body-site)		Oral, rectal, cervical, posterior vaginal fornix

For example, in the last row of the table, the project PRJDB10581 is linked to PMCID PMC8151423, from which the terms “Oral, rectal, cervical, posterior vaginal fornix” have been extracted to supplement the term “body-site” from the ENA record. Other examples include metadata about PCR primers (pcr_primers), marker genes (target_gene), nucleic acid extraction kit (nucl_acid_ext), body site (env_local_scale), environment phenomena (state), health or disease states (state), and treatment.

## Discussion

In this work, we provide a machine learning framework that enriches a wide variety of microbiome studies (19,209) with essential and accurate metadata from open access research articles (114,099). Using this framework, a new metagenomics annotations pipeline was developed and integrated into Europe PMC (Figure 2) to regularly enrich MGnify (Figure 3), with metadata for diverse metagenomics studies on an ongoing basis. MGnify uses the Europe PMC annotations API to retrieve metadata extracted from the literature for annotating data sets derived from host-associated (living organisms), ecoregion (natural environment), and engineered (humanmade) environments. To triage articles for curation, multiclass random forest models were trained to classify publications into these broad biome categories, which covered various and rich linguistic contexts for a wide range of organisms (e.g., mosquitoes, birds, chicken, ruminants, and humans), ecoregions (e.g., forests, oceans, rivers, lakes), and engineered environments (e.g., bioreactors, wastewater treatment plants, food, and microbial fuel cells) (see Supplementary Table S2). Subsequently, introducing 16 novel entities that flexibly accommodate the essential metadata of these environmental categories and training BioBERT NER models on their miscellaneous contexts have shown their utility for addressing the limitations of current databases and ontologies in describing extensive ranges of novel or hybrid microbiome environments.

Although metagenomics NER models demonstrated precisions of 80–100% (Table 3) for most of the entities, relatively lower F1-scores were observed with the categories engineered, site, state, and treatment. Qualitative assessment of these entities revealed that their metadata can be represented by single (unigrams) or multiple tokens (ngrams). Curators seemed to be more comprehensive in annotating entity-related tokens when compared to models, which tend to generate few yet precise annotations—hence, the relatively low token-wise recall (see Supplementary Table S9). Accordingly, low F1-scores seem not to necessarily reflect poor model performance but rather suggest that BERT-based models can perform better than curators in learning the contexts and patterns of weakly imprecise data [34]. Another limitation of the training data set is that the metadata of a small subset of entities (e.g., primer, place, and date) can be listed in publications tables, where rows and cells were merged and parsed as one sentence that might exceed the maximum sequence length (128) of model input. This resulted in the exclusion of tokens beyond maximum input length from being annotated by the model and subsequent low recall. On the other hand, training 16 individual NER models (one model per entity) provided considerable advantage for training overlapping entities, where each entity model has shown to require specific learning rate and training epochs to predict test data sets with higher precision. Alternatively, training one multiclass NER model on all entities was deemed to perform poorly due to the unbalanced BIO class distribution of single-class (nonoverlapping) versus multiclass (overlapping) entities, high number of trained entities (16), and small training data set. Moreover, modifying BERT tokenizer to keep and unsegment words with long lengths (e.g., primers) or unknown characters (e.g., kits, chemical states) managed to improve NER model performance, via retaining their representations by NER models instead of excluding them as unknown tokens (UNK) from input data. It is worth noting that pretraining BioBERT models on new PMC publications would have enabled the recognition of those unknown tokens during fine-tuning. But, due to the computationally expensive generation of contextual word representations from millions of publications, neither BioBERT models nor analogous neural architecture models, such as XLNet [55], RoBERTa [56], or ELECTRA [57], were pretrained or compared. These state-of-the-art models either outperformed BERT (e.g., XLNet) or showed comparable performance with less computing resources (e.g., RoBERTa and ELECTRA) on text classification, such as question answering, natural language inference, sentiment analysis, and document ranking, but not NER tasks. Future research will be needed to pretrain these models on millions of PubMed and PMC full-text articles and fine-tune them with metagenomics training data sets to explore whether masking tokens (BERT), permutation of token factorization order (XLNet), or replacing tokens with plausible alternatives (ELECTRA) could offer more accurate metagenomics annotations (NER) and address the complexity of novel metagenomics concepts. Overall, these training approaches and modifications have been shown to contribute to high prediction accuracy—hence, the implementation of metagenomics NER models in enriching the metadata of diverse microbiome studies in ENA and MGnify from Europe PMC publications.

We also demonstrated that the high accuracy of metagenomics NER models can extend to uncurated data sets. Training BioBERT-based NER models from scratch on large uncurated predicted data sets, generated from metagenomics NER models annotations, revealed the reversible transfer of their reliability and outperformance in predicting curated data sets with precision of 76–99% (see Supplementary Table S6). Moreover, evaluating the accuracy of model-derived annotations against ENA author-submitted metadata showed large overlaps between them in numerous studies regarding MIxS biome environmental packages, sequencing library strategy, material, location, host, sequencing methods, and collection dates (Figure 4). Conversely, although model-derived annotations showed greater MIxS coverage for more studies, limited overlaps were noticed due to incorrect, inaccurate, or inconsistent author-submitted data formats (see Supplementary Table S8). The analysis is not without limitations, as it covered metadata only from ENA fields used in more than 50 studies. However, it still offers some interesting insights. For instance, metagenomics annotations provided metadata for nearly 2,810 studies that had no sample or experimental metadata in ENA—hence, the enrichment of ambiguous sequence data sets to a more meaningful and exploitable data set. Examples of such cases include a study about the bacterial and fungal composition of soybean curd in tofu factories (PRJDB10470; PMC7563423) and the discovery of a new bacterial candidate phylum, *Candidatus kryptonia*, in Nevada hot springs (PRJEB11785; PMC4737851). In addition, exploring geographical metadata in ENA revealed that metagenomics annotations can enrich missing geographical locations for 7,541 studies, including 336 studies in MGnify. Examples from those studies are a study about taxonomic composition of soil viruses along the Namib Desert (PRJEB11968; MGYS00000581; PMC5256219) and a study about the bacterial community identified from sequencing a Neanderthal genome from a 38,000-year-old fossil (PRJEB1198; MGYS00000318; PMC3643900). Thus, enriching ENA with normalized model-predicted metadata is worth pursuing.

As demonstrated in Figure 4, this framework captures metadata terms that have a high degree of overlap to the corresponding metadata found in ENA, even when considering only exact terms. Moreover, the additional metadata identified through this framework will facilitate the more extensive use of existing data sets, which have been collected and sequenced at great expense and cannot simply be re-created. The expanded set of metadata will also simplify new meta-analyses, for example, the analysis of amplicon sequence variants (ASVs) [58], a method that can compare taxonomic profiles between different studies. However, ASV analysis requires that only equivalent regions are compared. Thus, the expansion of metadata related to amplicon data sets—namely, “target gene,” “target subfragment,” and “PCR primer”—means that data sets can now be rapidly selected using a single API call to Europe PMC, without the laboring reading of papers that would be otherwise be required. Note, that for very common terms like “rRNA,” it may be necessary to pair this with other terms, such as “library strategy” to ensure that the queries are restricted to amplicon-based studies. Furthermore, with the enriched metadata for shotgun metagenomics data sets, it will become easier to control for confounding factors by either producing closely paired data sets or statistically evaluating the significance of metadata differences. The wide range of metadata fields and biomes covered by the Europe PMC Submission System for Annotations means that these results are applicable to a broad range of researchers. This system is already linked to MGnify to enable the dynamic retrieval and display of additional metadata terms alongside users' submitted annotations. Given that MGnify provides a uniform analysis across databases, this eliminates confounding factors from bioinformatics analysis. Combined, the enriched metadata and uniform analyses will help eliminate many of the confounding factors that currently exist, making it easier to establish the link between compositional changes in the microbiota and phenotypic differences, as well as the identification of the underlying biological mechanism(s).

Lastly, given the breadth and variety of terms applicable to metagenomics, there is potential for using this pipeline to annotate other types of records in data resources such as BioStudies, BioSamples, and nucleotide sequences beyond metagenomics. Indeed, research articles that cite any accession number could be a target for “metagenomics" annotation with relevant entities (e.g., sample-material, sequencing, primer, place, date); the effectiveness of such an extension of this approach would need to be reevaluated to ensure accuracy and the general usefulness of the approach.

## Availability of Source Code

Project name: EMERALD

Project home page: https://gtr.ukri.org/projects?ref = BB%2FS009043%2F1

Operating system(s): Platform independent

Programming language: Python 3.7 or higher

Other requirements: genism 3.8.0, nltk 3.7, numpy 1.19.5, pandas 1.3.5, requests 2.27.1, scikit-learn 0.22.1, scispacy 0.5.0, spacy 3.0.8, tensorflow 1.15.0, tensorflow-gpu 1.15.0

License: Apache 2.0

Environment: Python with GPU/CPU support (3.7.3, miniconda 4.7.10)

EMERALD metagenomics annotations pipeline is available via GitLab at https://gitlab.com/maaly7/emerald_metagenomics_annotations and on BioTools (biotools:emerald_metagenomics_annotations_pipeline)

## Availability of Supporting Data

The data sets and models supporting the results of this article are publicly available via the GitLab repository and *GigaScience* database GigaDB [59]. The models were deposited in the BioModels EBI database (MODEL2202160002, MODEL2202170001-MODEL2202170015)

## Abbreviations

ASV: Amplicon Sequence Variant; BERT: Bidirectional Encoder Representations from Transformers; ENA: European Nucleotide Archive; ENVO: Environmental Ontology; GOLD: Genomes OnLine Database; LCM: Library Construction Method; LS: Library Strategy; MIxS: Minimum Information about any (x) Sequence; NER: Named Entity Recognition; PCR: Polymerase Chain Reaction (PCR); PMC: PubMed Central; rRNA: ribosomal RNA.

## Additional Files

**Table 1**: Random forest GOLD multiclass hierarchical levels

**Table 2**: Literature triage classified into 3 biome classes, using TF-IDF random forest model

**Table 3**: The performance metrics of TF-IDF biome classifiers when trained on abstract, introduction, method or discussion texts only. *classifiers trained on method sections showed the highest precision, recall and F1-score macro-averages.

**Table 4**: Queries used in retrieving metagenomics studies and publications from ENA, BioProject, Europe PMC, PMC and PubMed.

**Table 5**: The performance metrics of abstract, introduction, method, results and discussion sections classifiers. Classifiers features are either TF-IDF or Doc2VecENA (embeddings generated from training ENA cross-referenced publications). Support is the number of publications in test datasets per class.

**Table 6**: Token-wise precision, recall and F1-score macro-averages of the 16-best performance NER models trained on predicted datasets.

**Table 7**: Mapping ENA metadata fields and metagenomics entities to MIxS checklist

**Table 8**: Examples from matching ENA metadata with their metagenomics annotations counterparts. Cases for exact matches highlighted in bold (PRJDB3293, PRJDB8953, PRJEB11763, PRJDB10210), mismatches due inconsistent data synonyms or formats (PRJDB9252, PRJDB9200, PRJDB5495, PRJDB5860, PRJDB6715, PRJEB11804, PRJDB1602), missing ENA metadata (PRJDA73021, PRJDB8477, PRJDB9293, PRJDB5614, PRJDB7448, PRJDB8518, PRJEB11766, PRJEB11827), missing metagenomics annotations (PRJEB11799, PRJDB10112) and incorrect/inaccurate ENA metadata (PRJDB6461, PRJDB10611, PRJDB5850, PRJDB5860, PRJDB5936).

**Table 9**: Examples from test datasets showing entities models predictions versus curators annotations.

**Figure 1**: Receiver operating characteristic (ROC) curves of abstract (ABS), introduction(INTRO), methods (METHODS), results (RESULTS) and discussion (DISCUSS) sections classifiers, using TF-IDF (A) or Doc2VecENA (B) as training features.

**Curation guidelines**

giac077_GIGA-D-22-00054_Original_Submission

giac077_GIGA-D-22-00054_Revision_1

giac077_GIGA-D-22-00054_Revision_2

giac077_Response_to_Reviewer_Comments_Original_Submission

giac077_Response_to_Reviewer_Comments_Revision_1

giac077_Reviewer_1_Report_Original_Submission Levi D Waldron -- 4/12/2022 Reviewed

giac077_Reviewer_2_Report_Original_Submission Konstantinos Krampis, PhD -- 4/16/2022 Reviewed

giac077_Supplemental_File

## Competing Interests

The authors declare no competing interests.

## Funding

Francesco Talo' and Zunaira Shafique are funded by the Europe PMC grant, provided by 33 funders of life science research (https://europepmc.org/Funders/) under Wellcome Trust Grant 221523. Maaly Nassar and Santiago Sanchez were funded by Biotechnology and Biological Sciences Research Council (BB/S009043/1).

## Authors' Contributions

M.N. contributed to the study design and curation of training data sets, trained and validated the machine and deep learning models, developed the metagenomics annotations pipeline, analyzed the data, and wrote the manuscript. A.R. integrated metagenomics annotations into the MGnify database. F.T. and Z.S. integrated metagenomics annotations into the Europe PMC annotations platform. S.S. contributed to the curation and revision of training data sets. R.F. and J.M. conceived the project, contributed to the study design and contributed to and reviewed the manuscript.
