# Supplementary material for: A machine learning framework for discovery and enrichment of metagenomics metadata from open access publications
Source: Gigascience. 2022 Aug 11;11:giac077. doi: 10.1093/gigascience/giac077 (PMC9366992; doi:10.1093/gigascience/giac077)
Supplement: giac077_GIGA-D-22-00054_Revision_1 [file giac077_giga-d-22-00054_revision_1.pdf]

GigaScience

A machine learning framework for discovery and enrichment of metagenomics metadata from open access publications

--Manuscript Draft--

**Manuscript Number:**	GIGA-D-22-00054R1
**Full Title:**	A machine learning framework for discovery and enrichment of metagenomics metadata from open access publications
**Article Type:**	Research
**Funding Information:**	Biotechnology and Biological Sciences Research Council (BB/S009043/1) — Dr Robert D. Finn
**Abstract:**	Metagenomics is a culture-independent method for studying the microbes inhabiting a particular environment. Comparing the composition of samples (functionally/taxonomically), either from a longitudinal study or cross-sectional studies can provide clues into how the microbiota has adapted to the environment. However, a recurring challenge, especially when comparing results between independent studies, is that key metadata about the sample and molecular methods used to extract and sequence the genetic material are often missing from sequence records, making it difficult to account for confounding factors. Nevertheless, this missing metadata may be found in the narrative of publications describing the research. Here, we describe a machine learning framework that automatically extracts essential metadata for a wide range of metagenomics studies from the literature contained in Europe PMC. This framework has enabled the extraction of metadata from 114,099 publications in Europe PMC, including 19,900 publications describing metagenomics studies in ENA and MGnify.
**Corresponding Author:**	Jo McEntyre<br>EMBL-EBI: European Bioinformatics Institute<br>Cambridge, Cambs UNITED KINGDOM
**Corresponding Author Secondary Information:**	
**Corresponding Author's Institution:**	EMBL-EBI: European Bioinformatics Institute
**Corresponding Author's Secondary Institution:**	
**First Author:**	Maaly Nassar
**First Author Secondary Information:**	
**Order of Authors:**	Maaly Nassar
	Alexander Rogers
	Francesco Talo'
	Santiago Sanchez
	Zunaira Shafique
	Robert D. Finn
	Jo McEntyre
**Order of Authors Secondary Information:**	
**Response to Reviewers:**	Reviewer #1:<br>Figure 4 compares the annotations deposited by authors in the ENA to the authors' predictions from EPMC. This is a very useful comparison, and it would also be helpful for users of the published annotations, especially when there are non-identical or unique annotations. Are ENA annotations shown in addition to predicted annotations for users of the tools? I did see this feature mentioned in MGnify in the final Discussion paragraph, but I wasn't clear about within EPMC.

Powered by Editorial Manager® and ProduXion Manager® from Aries Systems Corporation

•The metadata terms found in EPMC are solely from the terms found within the article. While we appreciate that having additional metadata terms that a researcher may have submitted within the sequence record, there would not be a natural way of displaying this in EPMC, especially if the term was not found in EPMC. Similarly, ENA will not display metadata terms identified EPMC, as the records are owned by the submitter and cannot be modified. Thus, this functionality is performed by MGnify, but only on a study-by-study basis. As highlighted by the reviewer this comparison is useful but is restricted to this paper. Conceivably, it would be possible to recreate the analysis by using the respective resource APIs. We have tried to make this clearer in the manuscript.

Table 4: In the MIxS column it appears that the first term given is from MIxS, and the second term in parentheses is the authors' annotation term, but this isn't clear from the table or caption.

•MIxS (entity type) has been added to the MIxS column header.

The sentence "On the other hand, training 16 individual NER models (one model per entity) on all curated sentences provided extensive negative dataset and considerable advantage for training overlapping entities, …" is unclear, please clarify.

•We have tried to make this sentence clearer. More specifically, this sentence states that curating individual NER models addressed the overlapping entities issue. Furthermore, training each entity on all curated sentences, as mentioned in the methods, provided rich negative contexts. We deleted the negative context part to avoid confusion.

The pipeline continuously providing metagenomics annotations for new EPMC publications, and the user tools for accessing them, are mentioned almost only in passing in the Results. Many readers might miss this and think that all of this work was an interesting exercise but with results they can't readily use. Since the user tools built into EPMC, SciLite, and an API will be the part of greatest interest to many microbiome researchers, I recommend highlighting the availability of user tools in the Abstract, Introduction (which currently in my opinion has more than necessary on ML methods and is too brief on the final paragraph of how this work fills the previous gap), and Discussion (the mention in the Discussion that "This framework has been integrated into Europe PMC and further propagated to MGnify" does not make clear what this means to users).

•We appreciate this suggestion and have added additional sentences to the abstract and introduction to underscore how the algorithm/pipeline is used within EPMC and MGnify services and will be updated on a regular basis.

Reviewer #2:

Possibly a few sentences could be added to the discussion regarding application of the software beyond metagenomics (for example could it be applied to more broad annotation databases such as those of newly sequenced genomes).

•This is an interesting suggestion/comment. The reviewer is correct that there are many metadata terms that are identified by our models that would be equally applicable to other databases, such as nucleotide sequence records (where there will be the biggest overlap due to the use of the MIxS terms) that use other ‘omics techniques in sequencing biosamples beyond metagenomics. Conceivably, using the different biological resource accession numbers detected in EPMC, these models could be more widely applied to other articles and subsequently consumed by the source database providing the accession number. We have included some text at the end of the discussion to reflect this, but this would be beyond the scope of this article.

Powered by Editorial Manager® and ProduXion Manager® from Aries Systems Corporation

	Also a comment whether in the opinion of the authors other approaches for machine learning such as artificial neural networks / deep learning would yield different results than the approach used.<br><br>•We have included a brief description of other state-of-the-art models in the discussion section. These models were pretrained on wikipedia articles and have outperformed BERT or showed comparable performance in text classification tasks. However, they do not perform well on NER tasks. Fine-tuning these models with the metagenomics dataset is not expected to show better performance unless they were pretrained on a much larger training set than the one that was available to us (e.g., millions of PMC papers) as BioBERT and addressed the unknown token issue we have addressed in tensorflow BERT script. This is addressed in the discussion, where we have added further clarification. Also, it is worth noting that we have attempted to fine-tune BERT, XLNET, RoBERTa and ELECTRA models (pretrained on wikipedia) with metagenomics training dataset to explore if other model architectures will show better performance. And, initial performance metrics revealed BERT outperforming the other models. However, this was a preliminary fine-tuning attempt with wikipedia-pretrained models and an accurate model's comparison will require pretraining similar architecture models on millions of biomedical publications to address the complexity of novel metagenomics concepts (unknown tokens)
**Additional Information:**	
**Question**	**Response**
Are you submitting this manuscript to a special series or article collection?	No
**Experimental design and statistics**<br><br>Full details of the experimental design and statistical methods used should be given in the Methods section, as detailed in our Minimum Standards Reporting Checklist. Information essential to interpreting the data presented should be made available in the figure legends.<br><br>Have you included all the information requested in your manuscript?	Yes
**Resources**<br><br>A description of all resources used, including antibodies, cell lines, animals and software tools, with enough information to allow them to be uniquely identified, should be included in the Methods section. Authors are strongly encouraged to cite Research Resource Identifiers (RRIDs) for antibodies, model organisms and tools, where possible.	Yes

Have you included the information requested as detailed in our Minimum Standards Reporting Checklist?	
**Availability of data and materials**<br><br>All datasets and code on which the conclusions of the paper rely must be either included in your submission or deposited in publicly available repositories (where available and ethically appropriate), referencing such data using a unique identifier in the references and in the "Availability of Data and Materials" section of your manuscript.<br><br>Have you have met the above requirement as detailed in our Minimum Standards Reporting Checklist?	Yes

Manuscript

Click here to access/download;Manuscript;Nassar2022_revised-13-6-

A machine learning framework for discovery and enrichment of metagenomics metadata from open access publications

Maaly Nassar, Alexander B. Rogers, Francesco Talo', Santiago Sanchez, Zunaira Shafique, Robert D. Finn, Johanna McEntyre*

European Molecular Biology Laboratory, European Bioinformatics Institute (EMBL-EBI), Wellcome Trust Genome Campus, Hinxton, Cambridge CB10 1SD, UK.

* To whom correspondence should be addressed. Email: mcentyre@ebi.ac.uk

Abstract

Metagenomics is a culture-independent method for studying the microbes inhabiting a particular environment. Comparing the composition of samples (functionally/taxonomically), either from a longitudinal study or cross-sectional studies can provide clues into how the microbiota has adapted to the environment. However, a recurring challenge, especially when comparing results between independent studies, is that key metadata about the sample and molecular methods used to extract and sequence the genetic material are often missing from sequence records, making it difficult to account for confounding factors. Nevertheless, this missing metadata may be found in the narrative of publications describing the research. Here, we describe a machine learning framework that automatically extracts essential metadata for a wide range of metagenomics studies from the literature contained in Europe PMC. This framework has enabled the extraction of metadata from 114,099 publications in Europe PMC, including 19,900 publications describing metagenomics studies in ENA and MGnify. Using this framework, a new metagenomics annotations pipeline was developed and integrated into Europe PMC to regularly enrich up-to-date ENA and MGnify metagenomics studies with metadata extracted from research articles. These metadata are now available for researchers to explore and retrieve in the MGnify and Europe PMC websites, and Europe PMC annotations API.

Introduction

Whole genome shotgun DNA sequencing has emerged as a powerful tool for assessing the microbial content in a wide range of environmental samples. The advent of metagenomics analyses has enabled the phylogenetic and functional profiling of microbial communities found in a particular biome without the need of culturing. Meta-analysis between samples can reveal specific adaptations to environmental conditions, or candidate members of the community that may be responsible for health or disease [1–3]. However, to establish causal relationships between observed phenotypes and compositional changes, potential confounding factors such as individual alcohol consumption or therapeutic interventions need to be considered [4,5]. Thus, to undertake meaningful analyses spanning multiple metagenomics datasets, accurate contextual metadata about the environmental conditions from which the sample was taken and the experimental methods is required [6]. Despite the efforts to deposit, organise and analyse metagenomics data in public databases [7,8], major obstacles to their usability thereof remain, due to incomplete, missing and/or inaccurate contextual metadata.

Sequence databases, such as Sequence Read Archive (SRA) [9] and European Nucleotide Archive (ENA) [10], provide permanent storage for metagenome sequences, which offer the potential for knowledge databases, such as MGnify [11], MG-RAST [12] and Integrated Microbial Genomes with Microbiome Samples (IMG/M) [13], to explore the taxonomic diversity and metabolic pathways of biome microbial communities. Although significant advances have been made in the computation and indexing of metagenomics data, the lack of detailed and structured microbiome metadata has dramatically hampered metagenomics cross-study comparisons. To address this issue, metagenomics databases, such as Terrestrial Metagenome DB [14] , Human Microbiome Project [15] , Human Metagenome DB [16] , Genomes OnLine Database [17] (GOLD) and MGnify, have used controlled vocabularies from ontologies, such as ENVO (Environmental ontology) [18], to manually annotate a broad range of metagenomics samples. However, many of these studies have

incomplete detailed metadata about sample origins, due to the lack of metadata provided when depositing sequences into a database [19] and the inflexibility of hierarchical ontology relationships for describing diverse and specific environments, for example ENVO lacks vocabularies that could describe samples from many engineered environments. A scalable way to overcome these shortcomings is the development of automated methods to extract sample and experiment metadata from research articles describing metagenomics studies and link them to metagenomics datasets.

Named Entity Recognition (NER) is one of the foundational text-mining methods used for retrieving information from the literature. In life sciences, traditional NER approaches have relied on annotating vocabularies onto texts [20,21]. However, because of the limitation of their vocabularies, out-of-dictionary synonyms and restrictive hierarchical levels, current ontologies fail to capture essential data pertinent to diverse metagenomic studies; hence the urgent need for machine-guided approaches [22]. Consequently, hybrid machine and deep learning approaches, such as Bidirectional Long Short-Term Memory (BiLSTM) and Conditional Random Field (CRF) models, have been recently recognized to efficiently identify diverse entities in biomedical texts. Principally, these NER classifiers have relied on generating context-independent word representations (embeddings) from unlabelled literature corpora using word2vec language models [23,24], followed by the fine-tuning of these embeddings with hand-labelled data and task-specific neural architectures, such as LSTM- [25,26] or BiLSTM-CRF NER models [27–29]. But, whereas word2vec models aim to effectively capture the syntactic and semantic representations of words across diverse linguistic contexts, both context-independent and context-sensitive unidirectional representations [30] were found to be suboptimal for sentence- and token-classification tasks, where it is crucial to incorporate contexts from left and right directions (bidirectional representations) [31]. Hence, the emergence of the Bidirectional Encoder Representations from Transformers (BERT)-based models [32]. BERT alleviates both context-independent and context-sensitive unidirectional constraints with its unified architecture that can both

pretrain and fine-tune bidirectional context-sensitive word representations for diverse Natural Language Processing (NLP) tasks. BERT-pretrained word representations from English Wikipedia and Books Corpus managed to outperform hybrid architectures - context-independent or context-sensitive pretraining and BiLSTM-CRF fine-tuning models - in sentence- and word-classification (NER) tasks. Recently, BioBERT, a BERT-based model pretrained on PubMed abstracts and PMC full-text articles, has significantly outperformed BiLSTM-CRF and the original BERT models on most of the biomedical NER tasks [33]. Moreover, BioBERT has been recognized for its high performance in classifying medical entities after being fine-tuned with weakly supervised ontology-driven data [34].

Herein, we describe a machine-learning framework capable of enriching sample and experimental metadata of a wide range of metagenomics studies with terms extracted from open access publications in Europe PMC [35]. This framework: (1) classified and triaged publications describing research on diverse environments using machine learning models (literature classification), (2) constructed manually curated metagenomics training sets for 16 novel metagenomics entity types, (3) trained and validated BioBERT models to identify entities pertinent to the 16 metagenomics entity types in publications (NER), and (4) developed and integrated a fully automated metagenomics annotations pipeline that regularly enriches up-to-date metagenomics studies in MGnify and ENA and provide researchers with model-predicted metadata from research articles in Europe PMC (databases enrichments).

Material and Methods

Literature classification

Training dataset. Due to the lack of public datasets for training metagenomics-specific literature classifiers, we constructed our own training dataset based on MGnify, which provides access to a broad range of metagenomics studies and associated analysis results, but importantly curates microbiome studies with standardised biome annotations and

provides direct links to their relevant publications. Accordingly, a supervised training dataset was constructed by mapping the curated GOLD biome annotations assigned to the metagenomics studies in MGnify [36] to the corresponding publications in Europe PMC describing these studies (674 articles accessed on 17 February 2020). First, metagenomics studies were collected from MGnify with both PMC identifiers and GOLD annotations. Then, the full-text XML of publications corresponding to the PMC identifiers were retrieved with corresponding metadata from Europe PMC. Following XML preprocessing, sentences were parsed from the XML tags corresponding to each article section (i.e. introduction, method, results, discussion) and merged into individual section texts. Each text was aligned to the GOLD annotation of the corresponding metagenomic study and TF-IDF (Term Frequency-Inverse Document Frequency) or a vector of precomputed 200-dimensional Doc2Vec embedding [37] was used as features for biome classifiers. To generate Doc2Vec embeddings, publications cited in MGnify records were trained using the document-embedding neural network model Doc2Vec with the following parameters: 15 training iterations, a window size of 5, worker threads of 8 and a dimensional size of 200 (Python genism package 3.8: Doc2Vec).

Training random forest biome classifiers on cross-referenced publications. A total of 5 GOLD multiclass hierarchical levels (supplementary Table 1) were used to train nearly 50 random forest models (Python Scikit-learn package 0.22.1: RandomForestClassifier). For each of the TF-IDF and Doc2Vec approaches, a separate multiclass model was trained using the selected features for every hierarchical level. Each model trained a random selection of balanced datasets that comprised 20-100 texts per class. To validate model performance, datasets were divided into training datasets (80%) and test datasets (20%). The hyperparameter settings of random forest models were determined using a grid search among {50, 100, 150, 200, 250, 300} decision trees in the forest (n_estimators) and {25, 50, 75, 100, 125, 150, None} maximum tree depths (max_depth). Features selection was assessed through 5-fold shuffle-split cross-validation stratified by classes labels and random

state was fixed at model initialization for reproducibility. The precision (P), recall (R) and F1-score (F1) model performance metrics were compared and evaluated per hierarchical level.

***Biome classifiers prediction and implementation*.** A query was constructed to retrieve all publications associated with all metagenomics studies from the ENA database (determined by the descriptor "library source: METAGENOMIC" via ENA browser [38] and XREF APIs [39]). This resulted in 4513 publications from 9949 ENA metagenomics studies accessed on 20 February 2020. For each publication, full texts were then retrieved and processed using the same workflow employed in constructing the classifiers training dataset. The highest performing model with the broadest biome coverage was then selected to classify ENA cross-referenced publications. Using the model prediction probabilities, publications with probabilities of P >=0.4 were selected to have higher publications coverage per category with 70-95% precision. Lastly, a random sample of ENA cross-referenced publications that represents the breadth of biome classes (44-50 publications per class) were manually verified to validate the predicted biomes and subsequently used for the literature triage (supplementary Table 2).

Named Entity Recognition (NER)

Training dataset. A literature triage comprising 140 ENA cross-referenced publications were categorised into environmental (N=50), engineered (N=44), and host-associated (N=46) publications (supplementary Table 2). A total of 16 novel metagenomics entities, covering biome and experimental data, were proposed, and defined for biocuration (Table 1). Words pertinent to the aforementioned entities were curated in the publications validated for biocuration by a team of experienced curators, using Hypothes.is [40] - a web-based open-source annotation tool. Every manuscript was assigned to a single curator to annotate the entities under the supervision of another curator, followed by revisions from 2 different curators (see supplementary curation guidelines). After evaluation, annotations were retrieved via the Hypothesis API [41] and mapped to their corresponding sentences. Those sentences were then tokenized with BERT WordPiece tokenizer [42], which was modified to

retain words that are unknown to the BERT model (e.g., long primers, kits or chemical states with unknown characters), to be represented and trained by NER models instead of being excluded as unknown tokens (UNK). As entities can be nested within each other (overlapping entities), 16 individual training datasets were constructed to train each entity, separately. Each training dataset consisted of all curated sentences with their entity-related tokens tagged, by the standard NER classification scheme BIO [43], as the beginning (B), inside (I) or outside (O) of an entity.

Table 1: Metagenomics entity types

Entity	Definition
**Ecoregion**	Microbiome natural environment
**Host**	Microbiome living organism or host
**Engineered**	Microbiome man-made environment
**Date**	Microbiome sample collection date
**Place**	The place of microbiome environment or host
**Site**	The site of microbiome sample within place
**Body-Site**	The organ or tissue of microbiome sample
**Sample-Material**	The material of the microbiome sample (e.g., water, mucus, soil)
**State**	The state of microbiome environment or host (e.g., disease)
**Treatment**	Any treatment performed on the host (e.g., medicine) or the environment (e.g., fertiliser) from which the sample was collected
**Kit**	DNA extraction kit
**Primer**	PCR primers
**Gene**	Microbiome target genes, e.g., rRNA subunit and amplified region(s)
**LS**	Library source or library strategy (e.g., amplicon, whole genome)

**LCM**	Library construction method or layout (e.g., paired-end, single-end)
**Sequencing**	Sequencing platform

Training NER models on curated datasets. A total of 16 BioBERT models were trained to recognize metagenomics entities in publications, yielding 16 new metagenomics NER models. BioBERT pretrained weights generated from pre-training BERT on PubMed abstracts and PMC full texts were fine-tuned with each of the 16 training datasets. To validate the performance of each model, each dataset was divided into a training dataset (90%) and test dataset (10%). Fine-tuning was performed with a batch size of 32, maximum sequence length of 128 and fixed random state for reproducibility. A grid search was performed over 5 learning rates {1e-5, 2e-5, 3e-5, 4e-5} and 7 epochs {10,30,50,70,90,110,130} to select the highest performance hyperparameters for each model; thus, different hyperparameters for each training model. The token-wise macro-average precision (P), recall (R) and F1-score (F1) were compared and evaluated per each learning rate and epoch combination, separately.

NER models prediction and implementation. A total of 16 high-performance NER models (BioModels [44] accessions: MODEL2202160002, MODEL2202170001-MODEL2202170015) were used to annotate the methods sections of 114, 099 metagenomics publications. Since the best model performance metrics were observed when biome classifiers were trained on method texts (see supplementary Table 3), method sections were identified as the most representative article section for metagenomics data. The 114, 099 metagenomics publications were identified as follows: 1) cross-referencing metagenomics studies in genome and biosamples databases (ENA, NCBI Taxonomy, BioProject and MGnify) against literature repositories and 2) querying literature repositories (Europe PMC, PMC and PubMed) using metagenomics- and microbiome-related search and MeSH terms (for queries, see supplementary Table 4). Then, methods sections were

extracted from full-text XML via parsing the sentences wrapped within methods XML tags. Because metagenomics publications sometimes lack scientific methods sections, a multiclass random forest model was trained to classify full-text into abstract-, introduction-, methods-, results- and discussion-related texts. This section classifier (see supplementary Table 5 and Figure 1) was then used to extract methods-related text from publications lacking methods sections.

Training NER models on predicted datasets. To further demonstrate the accuracy of predicted annotations, a random subset of new metagenomics publications (1500 articles) was retrieved with their NER-models-predicted annotations to train new 16 NER BioBERT models from scratch. To construct this subset, trained biome classifiers were used to classify new metagenomics publications into 3 broad biome environments. Then, a random subset of 500 articles, with predicted probabilities of P >=0.5, was selected per biome class (i.e., engineered, environmental and host-associated classes), followed by the retrieval of their NER-models-predicted annotations. In this approach, those new publications with their NER-predicted annotations were considered as the training dataset, whereas the curated dataset was considered as the test dataset. This approach is like the student self-distillation process used in improving ImageNet classification [45] and protein structure prediction (AlphaFold) [46] models, where student models were trained on datasets predicted by teacher models (models trained on curated dataset). Though in our approach, it was hypothesised that teacher NER models - models trained on curated datasets - can generalise to novel biome publications, if students NER models - models trained on datasets predicted by teacher models - predicted curated datasets with token-wise macro-average F1-score >=70%.

Database enrichment

Europe PMC enrichment. Metagenomics annotations of 114, 099 publications in Europe PMC were represented in the annotations data model provided by the Europe PMC annotations submission system [47]. To make it easier to categorise and search publications with metagenomics annotations, specific metagenomics ontologies were required to

standardise the predicted annotations. However, with the surge in different environments being sampled, it became clear that metagenomics ontologies, including GOLD and ENVO, are insufficient for standardising most of the predicted annotations. Thus, ZOOMA [48], a semantic ontology mapping tool was used to map each of the predicted non-standardized annotations to a wide range of ontologies in the Ontology Lookup Service [49]. Processed annotations were then uploaded into the Europe PMC annotations API for users to search, explore and retrieve programmatically.

ENA and MGnify enrichment. Metagenomics annotations of publications describing 19,209 studies in ENA were amassed from the Europe PMC annotations API. To assess the enrichment of experimental and sample metadata, metagenomics annotations (NER-models-predicted annotations) were compared with the corresponding author-submitted metadata for 19,209 metagenomics studies in ENA. However, no sample or experimental metadata were associated with nearly 2810 studies in ENA. For each study, sample, experiment and run records identifiers - accessions - were parsed from ENA-SAMPLE, ENA-EXPERIMENT and ENA-RUN tags in the study XML, respectively, followed by the retrieval of run and experimental metadata from the identifier-associated records. Then, the entities of metagenomics annotations and a subset of ENA fields used in more than 50 studies were mapped to a corresponding subset of the minimum information about any (x) sequence (MIxS) [50] checklist (see supplementary Table 7). For each MIxS term, data were pooled from mapped ENA fields (author-submitted metadata) and metagenomics entities (metagenomics annotations) and compared per study. Studies with any identical or non-identical metadata in both sources, or having metadata from one source only were then counted per each MIxS term, with the results from the twenty most common MIxS fields shown in Figure 4.

Results

Overview

A machine learning framework was developed and integrated for enriching a wide variety of metagenomics studies with essential and accurate metadata from open access research articles. This framework: (1) trained machine learning models on cross-referenced data to classify a broad range of biome publications (literature classification and triage); (2) defined and curated 16 novel bio-entities that describe vital metadata for diverse metagenomics studies (biocuration; Table 1); (3) trained and validated BioBERT-based NER models on curated datasets (BioBERT fine-tuning and internal validation); (4) deployed 16 high performance trained NER models in retrieving accurate metagenomics metadata from thousands of publications in Europe PMC (NER); (5) standardised BioBERT-model-predicted annotations with multiple domain-specific ontologies (normalisation); (6) validated the transfer of models accuracy and reliability to uncurated datasets in Europe PMC and ENA (external validation); and lastly (7) developed an integrated and fully automated metagenomics annotations pipeline that regularly enriches up-to-date metagenomics studies (19,209) with model-predicted metadata from research articles (114,099) (databases enrichments).

Literature classification

Predicting a wide variety of microbiome environments was an essential requirement to triage publications for NER tasks. This requirement was addressed by constructing supervised training datasets, where the GOLD annotations assigned to metagenomics studies were mapped to the corresponding MGnify cross-referenced publications. Subsequently, random forest models were trained on the hierarchical levels of GOLD ontology yielding diverse biome prediction models. Comparing models performance metrics revealed higher F1-scores of 91-97% for the top GOLD hierarchical levels (Engineered, Environmental and Host-associated). However, Doc2Vec models were outperformed by their TF-IDF

alternatives (Table 2, Figure 1). Hence, TF-IDF models were applied for the prediction of microbiome environments in new metagenomics publications (selected based on the publication being referenced from an ENA study record) and subsequently literature triage.

Table 2. Precision, Recall and F1-scores of the best performance random-forest biome classifiers.

Classifier features	Class	Precision	Recall	F1-Score	Support
**TF-IDF**	Engineered	0.86	0.9	0.88*	20
	Environmental	0.86	0.9	0.88*	20
	Host-associated	0.94	0.85	0.89*	20
**Doc2Vec^{MGnify}**	Engineered	0.77	0.85	0.81	20
	Environmental	0.89	0.8	0.84	20
	Host-associated	0.85	0.85	0.85	20

Classifiers features were either TF-IDF or Doc2Vec^{MGnify} (embeddings generated from MGnify cross-referenced publications). Support = number of publications in test datasets per class. *TF-IDF biome classifiers outperformed the ones trained on Doc2Vec^{MGnify}. n_estimators=300 (TF-IDF) and 250 (Doc2Vec^{MGnify}). max_depth = 25. Random state = 9.

Figure 1: Receiver operating characteristic (ROC) curves of biome classifiers, using TF-IDF (A) or Doc2Vec^{MGnify} (B) as training features.

Named Entity Recognition (NER)

To identify metagenomics data in research articles, 16 novel metagenomics entities (Table 1) were defined, curated, and trained using NER models. Training and test datasets were constructed from the curated 140 ENA cross-referenced publications, which were not contained in the MGnify database to ensure unbiased and broader coverage of biome. Triaged publications (see supplementary Table 2) were randomly selected and equally categorised using highest performance random forest classifiers (Table 2, Figure 1). The manual curation of metagenomics entities in literature triage yielded 9567 annotations for 2496 sentences. As entities can be nested within each other, 16 individual datasets were constructed separately for fine-tuning 16 BioBERT models. Each entity dataset, comprising

2496 BERT-tokenized sentences with BIO-tagged entities, was partitioned for training (90%; 2246 sentences) and testing (10%; 250 sentences). During training, grid search over 5 learning rates and 7 epochs showed best performance models with diverse combinations of hyperparameters per entity. These varieties of combinations were expected to give the best performance, given the overlapping nature of the entities. Typically, the greater the contexts overlap, the greater the training the model required to achieve the best performance. Table 3 shows the best token-wise precision (P), recall (R) and F1-score (F1) macro-averages for trained NER models per entity. Models achieved a precision of 80-100% and a F1-score between 71-98%.

Table 3: Token-wise precision, recall and F1-score of the 16-best performance NER models.

Entity	Learning Rate	Epoch	Recall	Precision	F1-Score
**Ecoregion**	4e-5	50	0.95	1	0.98
**Host**	2e-5	90	0.89	0.93	0.9
**Engineered**	2e-5	10	0.65	0.93	0.75
**Date**	4e-5	90	0.78	0.91	0.83
**Place**	3e-5	90	0.78	0.86	0.82
**Site**	4e-5	10	0.71	0.85	0.77
**Body-Site**	4e-5	90	0.98	0.95	0.97
**Sample-Material**	5e-5	110	0.8	0.9	0.85
**State**	5e-5	110	0.65	0.8	0.71
**Treatment**	4e-5	30	0.66	0.8	0.73
**Kit**	2e-5	70	0.94	0.91	0.92
**Primer**	5e-5	70	0.94	0.97	0.96
**Gene**	1e-5	10	0.86	0.92	0.89
**LS**	5e-5	50	0.8	0.95	0.85

**LCM**	4e-5	50	0.86	1	0.92
**Sequencing**	5e-5	110	0.84	0.89	0.87

To validate the accuracy of NER models predictions, 16 new NER models were trained from scratch on uncurated predicted annotations from 1500 new articles. The trained models were then tested in recognizing entities in the curated datasets (140 articles that were not included in the 1500 training articles). Table 6 (supplementary) shows the best token-wise P, R and F1 macro-averages for the NER models trained on predicted entities, where F1-scores were 73-97%. These NER models outperformed those trained on the curated datasets for *date, place, state, treatment, kit, gene, LS, LCM* and *sequencing* entities.

Database enrichment

To enrich metagenomics studies with data from research articles, 114,099 publications in Europe PMC were processed and annotated using the highest NER performance models (Table 3). Those publications encompassed 19,900 publications linked to studies in the ENA and MGnify databases.

A new pipeline was developed to continuously provide metagenomics annotations for new open access publications. Users are now allowed to search, explore and retrieve metagenomics annotations programmatically from the Europe PMC website, using search queries (e.g. ANNOTATION_PROVIDER:"Metagenomics" [51]), a SciLite application (Figure 2 : article view) and the annotations API [52], respectively. The pipeline was tested on Linux operating system and Google Colab GPUs and CPUs. A minimum of Python 3.6 or 3.7 is required. In addition, if Conda will be used as python packages manager, then a minimum version of Miniconda 4.7.10 can be installed to create Python 3.7 environment with all the necessary packages. A more detailed description about the necessary packages can be found under “Availability of Source Code” section and the project Gitlab repository.

Figure 2: Screenshot of Europe PMC article. Annotations panel with metagenomics entities and annotations (right). Highlighted annotations in full-text using SciLite tool (left) (SciLite article view from PMC8791192).

To date, MGnify has enriched the metadata of 2310 studies with the metagenomics annotations from corresponding articles (1800) using the Europe PMC annotations API. Enriched metadata were made accessible in MGnify publications [53] and sample web pages (Figure 3: study view) for researchers to explore in metagenomics analyses. Moreover, a total of 1,658,023 of metagenomics annotations were linked to BioSamples records and deposited in ELIXIR Contextual Data Clearinghouse (under curations and providerName=EMERALD) [54] for curators to validate and integrate into ENA.

Figure 3: Composite screenshot showing annotations-enriched metadata for a MGnify study. Annotated Publications are highlighted within a Study (left) and annotations are shown in context (right).

To further evaluate our approach, the publication-derived metagenomics data were compared against author-submitted metadata for 19,209 studies. Both sets of metadata were mapped to the relevant MIxS checklist, allowing the annotations to be compared. Since both publication-derived metagenomics data and author-submitted metadata were provided as free text, comparing them was a non-trivial task; hence, the comparison of exact matches. This comparison revealed that for the vast majority of MIxS checklist terms, the numbers of publication-derived terms were greater than the numbers retrieved from ENA author-submitted metadata, Figure 4 (see supplementary Figure 4 Data, Figure 4 Analysis Data). In addition, metagenomics annotations demonstrated high quality coverage for many of the missing metadata in ENA, such as PCR primers (pcr_primers), phylogenetic marker genes (target_gene), nucleic acid extraction kit (nucl_acid_ext), environment phenomena (state), health or disease states (state) and treatment (Table 4). Moreover, overlaps were observed between metagenomics annotations and ENA metadata in numerous studies, regarding MIxS env_package (12,062 studies), lib_layout (8173 studies), env_medium

(7,058 studies), source_uvig (5,789 studies), geo_loc_name (5,445 studies), specific_host (2,597 studies), host_spec_range (2,597 studies), env_local_scale (2,412 studies), seq_meth (1,649 studies), collection_date (1,002 studies), target_subfragment (241 studies), env_broad_scale (144 studies), health_disease_stat (126 studies), depth (120 studies), lat_lon (91 studies), target_gene (80 studies), pcr_primers (58 studies), elev (26 studies), alt (2 studies) and nucl_acid_ext (1 study). Yet, many studies have shown no or limited overlap, either due to missing essential metadata or having inaccurate, incorrect or inconsistent metadata (i.e. synonyms or unit formats) in ENA compared with their metagenomics annotations counterparts (see supplementary Table 8).

Figure 4: A clustered stacked bar plot showing the overlap of metadata terms between ENA (author submitted terms that are part of the study) and those derived from Europe PMC (EPMC) articles using our framework. For each study, we have evaluated twenty of the most populated MIxS fields in 19,209 studies, to establish if the metadata provided by both sources were identical (green), non-identical (yellow), or provided by one of the metadata sources only (unique, red). For each MIxS field, each stacked bar shows the number of studies having identical, non-identical and unique metadata from each source.

Table 4. This table provides some examples of how the metagenomics annotations extracted from the publications describing ENA records enrich the metadata for those studies.

Study	PMCID	MIxS (Entity Type)	ENA Metadata	Metagenomics Annotations
PRJDB8863	PMC6941062	pcr_primers (primer)	-	RNA (rRNA) genes (forward: 5′-ACACTCTTTCCCTACACGACGCTCTTCCGATCTGTGCCAGCMGCCGCGGTAA-3′; reverse: 5′-GTGACTGGAGTTCAGAC

				GTGTGCTCTTCCGATCT GGACTACHVGGGTWTC TAAT-3'
PRJDB9293	PMC8147061	target_gene (gene)	-	16S rRNA, V3-V4
PRJDB5614	PMC5745017	nucl_acid_ext (kit)	-	RNA PowerSoil total RNA isolation kit
PRJEB27411	PMC6072794	Env_package (state)	-	100-year drought, 75.6 mm of rainfall, mesotrophic, denitrification
PRJEB15392	PMC5405060	health_disease_stat (state)	-	caries, gingivitis, medically healthy, oropharyngeal mucositis, poor oral hygiene, pulpal diseases
PRJEB22207	PMC7044117	Env_package (treatment)	-	Antibiotic, benzylpenicillin, cefotaxime, gentamicin, meropenem, metronidazole, probiotic supplementation, vancomycin
PRJDB10581	PMC8151423	env_local_scale (body-site)	-	Oral, Rectal, cervical, posterior vaginal fornix

For example, in the last row of the table, the project PRJD810581 is linked to PMCID PMC8151423, from which the terms “Oral, Rectal, cervical, posterior vaginal fornix” have been extracted to supplement the term “body-site” from the ENA record. Other examples include metadata about PCR

primers (pcr_primers), marker genes (target_gene), nucleic acid extraction kit (nucl_acid_ext), body site (env_local_scale), environment phenomena (state), health or disease states (state) and treatment.

Discussion

In this work, we provide a machine learning (ML) framework that enriches a wide variety of microbiome studies (19,209) with essential and accurate metadata from open access research articles (114,099). Using this framework, a new metagenomics annotations pipeline was developed and integrated into Europe PMC (Figure 2) to regularly enrich MGnify (Figure 3), with metadata for diverse metagenomics studies on an ongoing basis. MGnify uses the Europe PMC annotations API to retrieve metadata extracted from the literature for annotating datasets derived from host-associated (living organisms), ecoregion (natural environment) and engineered (man-made) environments. To triage articles for curation, multiclass random forest models were trained to classify publications into these broad biome categories, which covered various and rich linguistic contexts for a wide range of organisms (e.g., mosquitoes, birds, chicken, ruminants and human), ecoregions (e.g., forests, oceans, rivers, lakes) and engineered environments (e.g., bioreactors, wastewater treatment plants, food, and microbial fuel cells) (see supplementary Table 2). Subsequently, introducing 16 novel entities that flexibly accommodate the essential metadata of these environmental categories and training BioBERT NER models on their miscellaneous contexts have shown their utility for addressing the limitations of current databases and ontologies in describing extensive ranges of novel or hybrid microbiome environments.

Although metagenomics NER models demonstrated precisions of 80-100% (Table 3) for most of the entities, relatively lower F1-scores were observed with the categories engineered, site, state and treatment. Qualitative assessment of these entities revealed that their metadata can be represented by single (unigrams) or multiple tokens (ngrams). Curators seemed to be more comprehensive in annotating entity-related tokens when compared to models, which tend to generate few, yet precise annotations; hence, the relatively low token-wise recall (see supplementary Table 9). Accordingly, low F1-scores

seem not necessarily reflecting poor model performance, but rather suggest that BERT-based models can perform better than curators in learning the contexts and patterns of weakly imprecise data [34]. Another limitation of the training dataset is that the metadata of a small subset of entities (e.g., primer, place and date) can be listed in publications tables, where rows and cells were merged and parsed as one sentence that might exceed the maximum sequence length (128) of model input. This resulted in the exclusion of tokens beyond maximum input length from being annotated by the model and subsequent low recall. On the other hand, training 16 individual NER models (one model per entity) provided considerable advantage for training overlapping entities, where each entity model has shown to require specific learning rate and training epochs to predict test datasets with higher precision. Alternatively, training one multiclass NER model on all entities was deemed to perform poorly due to the unbalanced BIO class distribution of single- (non-overlapping) versus multiclass (overlapping) entities, high number of trained entities (16) and small training dataset. Moreover, modifying BERT tokenizer to keep and unsegment words with long lengths (e.g., primers) or unknown characters (e.g., kits, chemical states) managed to improve NER model performance, via retaining their representations by NER models instead of excluding them as unknown tokens (UNK) from input data. It is worth noting that pretraining BioBERT models on new PMC publications would have enabled the recognition of those unknown tokens during fine-tuning. But, due to the computationally expensive generation of contextual word representations from millions of publications, neither BioBERT models or analogous neural architecture models, such as XLNet [55], RoBERTa [56] or ELECTRA [57], were pre-trained or compared. These state-of-the-art models either outperformed BERT (e.g. XLNet) or showed comparable performance with less computing resources (e.g. RoBERTa and ELECTRA) on text classification, such as question answering, natural language inference, sentiment analysis, and document ranking, but not NER tasks. Future research will be needed to pretrain these models on millions of PubMed and PMC full-text articles and fine-tune them with metagenomics training datasets to explore whether masking tokens (BERT), permutation of tokens factorization order (XLNet) or replacing

tokens with plausible alternatives (ELECTRA) could offer more accurate metagenomics annotations (NER) and address the complexity of novel metagenomics concepts. Overall, these training approaches and modifications have been shown to contribute to high prediction accuracy; hence, the implementation of metagenomics NER models in enriching the metadata of diverse microbiome studies in ENA and MGnify from Europe PMC publications.

We also demonstrated that the high accuracy of metagenomics NER models can extend to uncurated datasets. Training BioBERT-based NER models from scratch on large uncurated predicted datasets, generated from metagenomics NER models annotations, revealed the reversible transfer of their reliability and outperformance in predicting curated datasets with precision of 76-99% (see supplementary Table 6). Moreover, evaluating the accuracy of model-derived annotations against ENA author-submitted metadata showed large overlaps between them in numerous studies regarding MIxS biome environmental packages, sequencing library strategy, material, location, host, sequencing methods, and collection dates (Figure 4). Conversely, although model-derived annotations showed greater MIxS coverage for more studies, limited overlaps were noticed due to incorrect, inaccurate, or inconsistent author-submitted data formats (see supplementary Table 8). The analysis is not without limitations, as it covered metadata only from ENA fields used in more than 50 studies. However, it still offers some interesting insights. For instance, metagenomics annotations provided metadata for nearly 2810 studies that had no sample or experimental metadata in ENA; hence the enrichment of ambiguous sequence datasets to a more meaningful and exploitable dataset. Examples of such cases include a study about the bacterial and fungal composition of soybean curd in tofu factories (PRJDB10470; PMC7563423) and the discovery of new bacterial candidate phylum, *Candidatus Kryptonia*, in Nevada hot springs (PRJEB11785; PMC4737851). In addition, exploring geographical metadata in ENA revealed that metagenomics annotations can enrich missing geographical locations for 7541 studies, including 336 studies in MGnify. Examples from those studies are

a study about taxonomic composition of soil viruses along the Namib Desert (PRJEB11968; MGYS00000581; PMC5256219) and a study about the bacterial community identified from sequencing neanderthal genome from a 38,000-year-old fossil (PRJEB1198; MGYS00000318; PMC3643900). Thus, enriching ENA with normalised model-predicted metadata is worth pursuing.

As demonstrated in Figure 4, this framework captures metadata terms that have a high degree of overlap to the corresponding metadata found in ENA, even when considering only exact terms. Moreover, the additional metadata identified through this framework will facilitate the more extensive use of existing datasets, which have been collected and sequenced at great expense and cannot simply be recreated. The expanded set of metadata will also simplify new meta-analyses, for example the analysis of amplicon sequence variants (ASVs) [58], a method that can compare taxonomic profiles between different studies. However, ASV analysis requires that only equivalent regions are compared. Thus, the expansion of metadata related to amplicon datasets, namely "target gene", "target subfragment" and "PCR primer", means that datasets can now be rapidly selected using a single API call to Europe PMC, without the labouring reading of papers that would be otherwise be required. Note, that for very common terms like "rRNA", it may be necessary to pair this with other terms, such as "library strategy" to ensure that the queries are restricted to amplicon-based studies. Furthermore, with the enriched metadata for shotgun metagenomics datasets, it will become easier to control for confounding factors by either producing closely paired datasets or statistically evaluating the significance of metadata differences. The wide range of metadata fields and biomes covered by the Europe PMC Submission System for Annotations means that these results are applicable to a broad range of researchers. This system is already linked to MGnify to enable the dynamic retrieval and display of additional metadata terms alongside users submitted annotations. Given that MGnify provides a uniform analysis across databases, this eliminates confounding factors from bioinformatics analysis. Combined, the enriched metadata and

uniform analyses will help eliminate many of the confounding factors that currently exist, making it easier to establish the link between compositional changes in the microbiota and phenotypic differences, and the identification of the underlying biological mechanism(s).

Lastly, given the breadth and variety of terms applicable to metagenomics, there is potential for using this pipeline to annotate other types of records in data resources such as BioStudies, and BioSamples, and nucleotide sequences beyond metagenomics. Indeed, research articles that cite any accession number could be a target for “metagenomics" annotation with relevant entities (e.g. sample-material, sequencing, primer, place, date); the effectiveness of such an extension of this approach would need to be re-evaluated to ensure accuracy and the general usefulness of the approach.

Availability of Source Code

Project name: EMERALD

Project home page: https://gitlab.com/maaly7/emerald_metagenomics_annotations

Operating system(s): Platform independent

Programming language: Python 3.7 or higher

Other requirements: genism 3.8.0, nltk 3.7, numpy 1.19.5, pandas 1.3.5, requests 2.27.1, scikit-learn 0.22.1, scispacy 0.5.0, spacy 3.0.8, tensorflow 1.15.0, tensorflow-gpu 1.15.0,

License: Apache 2.0

Environment: Python with GPU/CPU support (3.7.3, miniconda 4.7.10)

The data set(s) supporting the results of this article is available in the above GitLab repository and the models are available in BioModels EBI database [MODEL2202160002, MODEL2202170001-MODEL2202170015)].

Acknowledgement

The authors would like to thank the curators from Molecular Connections (https://www.molecularconnections.com/) for their biocuration efforts, Zhan Huang and

Lynne Faulk (EMBL-EBI) for user interface design and implementation, Audrey Hamelers (EMBL-EBI) for proofreading the manuscript, and Awais Athar and Santosh Tirunagari (EMBL-EBI) for testing and validating the Gitlab repository.

Conflict of interest

The authors declare no competing interests

Funding

Francesco Talo' and Zunaira Shafique are funded by the Europe PMC grant, provided by 33 funders of life science research (https://europepmc.org/Funders/) under Wellcome Trust Grant 221523. Maaly Nassar and Santiago Sanchez were funded by Biotechnology and Biological Sciences Research Council (BB/S009043/1).

Contributions

Maaly Nassar contributed to the study design and curation of training datasets, trained and validated the machine and deep learning models, developed the metagenomics annotations pipeline, analysed the data and wrote the manuscript. Alexander Rogers integrated metagenomics annotations into the MGnify database. Francesco Talo' and Zunaira Shafique integrated metagenomics annotations into the Europe PMC annotations platform. Santiago Sanchez contributed to the curation and revision of training datasets. Robert Finn and Johanna McEntyre conceived the project, contributed to the study design and contributed to, and reviewed, the manuscript.

References

1. Reitmeier S, Kiessling S, Clavel T, List M, Almeida EL, Ghosh TS, et al.. Arrhythmic Gut Microbiome Signatures Predict Risk of Type 2 Diabetes. *Cell Host & Microbe*. Cell Press; 2020; doi: 10.1016/J.CHOM.2020.06.004.
2. Oh TG, Kim SM, Caussy C, Fu T, Guo J, Bassirian S, et al.. A Universal Gut-Microbiome-Derived Signature Predicts Cirrhosis. *Cell Metabolism*. Cell Press; 2020; doi: 10.1016/J.CMET.2020.06.005.
3. Weber L, González- Díaz P, Armenteros M, Ferrer VM, Bretos F, Bartels E, et al.. Microbial signatures of protected and impacted Northern Caribbean reefs: changes from Cuba to the Florida Keys. *Environmental Microbiology*. Wiley-Blackwell; 2020; doi: 10.1111/1462-2920.14870.
4. Forslund K, Hildebrand F, Nielsen T, Falony G, le Chatelier E, Sunagawa S, et al.. Disentangling type 2 diabetes and metformin treatment signatures in the human gut microbiota. *Nature 2015 528:7581*. Nature Publishing Group; 2015; doi: 10.1038/nature15766.
5. Vujkovic-Cvijin I, Sklar J, Jiang L, Natarajan L, Knight R, Belkaid Y. Host variables confound gut microbiota studies of human disease. *Nature 2020 587:7834*. Nature Publishing Group; 2020; doi: 10.1038/s41586-020-2881-9.
6. Nayfach S, Pollard KS. Toward Accurate and Quantitative Comparative Metagenomics. *Cell*. Cell Press; 2016; doi: 10.1016/J.CELL.2016.08.007.
7. Mirzayi C, Renson A, Furlanello C, Sansone SA, Zohra F, Elsafoury S, et al.. Reporting guidelines for human microbiome research: the STORMS checklist. *Nature Medicine 2021 27:11*. Nature Publishing Group; 2021; doi: 10.1038/s41591-021-01552-x.
8. Yilmaz P, Gilbert JA, Knight R, Amaral-Zettler L, Karsch-Mizrachi I, Cochrane G, et al.. The genomic standards consortium: bringing standards to life for microbial ecology. *The ISME Journal*. 2011; doi: 10.1038/ISMEJ.2011.39.
9. Kodama Y, Shumway M, Leinonen R. The sequence read archive: explosive growth of sequencing data. *Nucleic Acids Research*. Oxford University Press; 2012; doi: 10.1093/NAR/GKR854.
10. Harrison PW, Ahamed A, Aslam R, Alako BTF, Burgin J, Buso N, et al.. The European Nucleotide Archive in 2020. *Nucleic Acids Research*. Oxford University Press; 2021; doi: 10.1093/NAR/GKAA1028.
11. Mitchell AL, Scheremetjew M, Denise H, Potter S, Tarkowska A, Qureshi M, et al.. EBI Metagenomics in 2017: enriching the analysis of microbial communities, from sequence reads to assemblies. *Nucleic Acids Research*. Oxford University Press; 2018; doi: 10.1093/NAR/GKX967.
12. Wilke A, Bischof J, Gerlach W, Glass E, Harrison T, Keegan KP, et al.. The MG-RAST metagenomics database and portal in 2015. *Nucleic Acids Research*. Oxford University Press; 2016; doi: 10.1093/NAR/GKV1322.
13. Chen I-MA, Markowitz VM, Chu K, Palaniappan K, Szeto E, Pillay M, et al.. IMG/M: integrated genome and metagenome comparative data analysis system. *Nucleic Acids Research*. Oxford University Press; 2017; doi: 10.1093/NAR/GKW929.
14. Corrêa FB, Saraiva JP, Stadler PF, da Rocha UN. TerrestrialMetagenomeDB: a public repository of curated and standardized metadata for terrestrial metagenomes. *Nucleic acids research*. 2020; doi: 10.1093/nar/gkz994.

15. Proctor LM, Creasy HH, Fettweis JM, Lloyd-Price J, Mahurkar A, Zhou W, et al.. The Integrative Human Microbiome Project. *Nature*. Nature Publishing Group; 2019; doi: 10.1038/s41586-019-1238-8.
16. Kasmanas JC, Bartholomäus A, Corrêa FB, Tal T, Jehmlich N, Herberth G, et al.. HumanMetagenomeDB: a public repository of curated and standardized metadata for human metagenomes. *Nucleic Acids Research*. Oxford University Press; 2021; doi: 10.1093/NAR/GKAA1031.
17. Mukherjee S, Stamatis D, Bertsch J, Ovchinnikova G, Sundaramurthi JC, Lee J, et al.. Genomes OnLine Database (GOLD) v.8: Overview and updates. Nucleic Acids Research. Oxford University Press;
18. Buttigieg PL, Morrison N, Smith B, Mungall CJ, Lewis SE. The environment ontology: Contextualising biological and biomedical entities. *Journal of Biomedical Semantics*. BioMed Central Ltd.; 2013; doi: 10.1186/2041-1480-4-43.
19. Bagheri H, Severin AJ, Rajan H. Detecting and correcting misclassified sequences in the large-scale public databases. *Bioinformatics*. Oxford Academic; 2020; doi: 10.1093/BIOINFORMATICS/BTAA586.
20. Pappas KM, Tassou C, Venieri D, Zafeiropoulos H, Paragkamian S, Ninidakis S, et al.. PREGO: A Literature and Data-Mining Resource to Associate Microorganisms, Biological Processes, and Environment Types. *Microorganisms 2022, Vol 10, Page 293*. Multidisciplinary Digital Publishing Institute; 2022; doi: 10.3390/MICROORGANISMS10020293.
21. Chaix E, Deléger L, Bossy R, Nédellec C. Text mining tools for extracting information about microbial biodiversity in food. *Food Microbiology*. Elsevier; 2019; doi: 10.1016/J.FM.2018.04.011.
22. Arighi CN, Wu CH, Cohen KB, Hirschman L, Krallinger M, Valencia A, et al.. BioCreative-IV virtual issue. *Database: The Journal of Biological Databases and Curation*. Oxford University Press; 2014; doi: 10.1093/DATABASE/BAU039.
23. Mikolov T, Sutskever I, Chen K, Corrado G, Dean J. Distributed Representations of Words and Phrases and their Compositionality. *Advances in Neural Information Processing Systems*. Neural information processing systems foundation; 2013;
24. Mikolov T, Chen K, Corrado G, Dean J. Efficient Estimation of Word Representations in Vector Space. *1st International Conference on Learning Representations, ICLR 2013 - Workshop Track Proceedings*. International Conference on Learning Representations, ICLR; 2013;
25. Habibi M, Weber L, Neves M, Wiegandt DL, Leser U. Deep learning with word embeddings improves biomedical named entity recognition. *Bioinformatics*. Oxford University Press; 2017; doi: 10.1093/BIOINFORMATICS/BTX228.
26. Giorgi JM, Bader GD. Transfer learning for biomedical named entity recognition with neural networks. *Bioinformatics*. Oxford University Press; 2018; doi: 10.1093/BIOINFORMATICS/BTY449.
27. Luo L, Yang Z, Yang P, Zhang Y, Wang L, Lin H, et al.. An attention-based BiLSTM-CRF approach to document-level chemical named entity recognition. *Bioinformatics*. Oxford Academic; 2018; doi: 10.1093/BIOINFORMATICS/BTX761.
28. Dang TH, Le H-Q, Nguyen TM, Vu ST. D3NER: biomedical named entity recognition using CRF-biLSTM improved with fine-tuned embeddings of various linguistic information. Wren J, editor. *Bioinformatics*. Narnia; 2018; doi: 10.1093/bioinformatics/bty356.

29. Xu K, Yang Z, Kang P, Wang Q, Liu W. Document-level attention-based BiLSTM-CRF incorporating disease dictionary for disease named entity recognition. *Computers in Biology and Medicine*. Pergamon; 2019; doi: 10.1016/J.COMPBIOMED.2019.04.002.
30. Vaswani A, Shazeer N, Parmar N, Uszkoreit J, Jones L, Gomez AN, et al.. Attention is all you need. *Advances in Neural Information Processing Systems*. Neural information processing systems foundation; p. 5999–6009.
31. Peters ME, Neumann M, Iyyer M, Gardner M, Clark C, Lee K, et al.. Deep Contextualized Word Representations. *NAACL HLT 2018 - 2018 Conference of the North American Chapter of the Association for Computational Linguistics: Human Language Technologies - Proceedings of the Conference*. Association for Computational Linguistics (ACL); 2018; doi: 10.18653/V1/N18-1202.
32. Devlin Google J, Language AI. BERT: Pre-training of Deep Bidirectional Transformers for Language Understanding (Bidirectional Encoder Representations from Transformers).
33. Lee J, Yoon W, Kim S, Kim D, Kim S, So CH, et al.. BioBERT: a pre-trained biomedical language representation model for biomedical text mining. Wren J, editor. *Bioinformatics*. 2019; doi: 10.1093/bioinformatics/btz682.
34. Fries JA, Steinberg E, Khattar S, Fleming SL, Posada J, Callahan A, et al.. Ontology-driven weak supervision for clinical entity classification in electronic health records. *Nature Communications 2021 12:1*. Nature Publishing Group; 2021; doi: 10.1038/s41467-021-22328-4.
35. Ferguson C, Araújo D, Faulk L, Gou Y, Hamelers A, Huang Z, et al.. Europe PMC in 2020. *Nucleic acids research*. NLM (Medline); 2021; doi: 10.1093/nar/gkaa994.
36. : Home < MGnify < EMBL-EBI. https://www.ebi.ac.uk/metagenomics/ Accessed 2022 Mar 14.
37. Le Q v., Mikolov T. Distributed Representations of Sentences and Documents. *31st International Conference on Machine Learning, ICML 2014*. International Machine Learning Society (IMLS); 4:2931–92014;
38. : ENA Browser. https://www.ebi.ac.uk/ena/browser/home Accessed 2022 Mar 14.
39. : ENA Xref. https://www.ebi.ac.uk/ena/xref/rest/#!/JSON/getSourcesUsingGET Accessed 2022 Mar 14.
40. : Home : Hypothesis. https://web.hypothes.is/ Accessed 2022 Mar 14.
41. : Hypothesis API documentation (v1). https://h.readthedocs.io/en/latest/api-reference/ Accessed 2022 Mar 14.
42. Wu Y, Schuster M, Chen Z, Le Q v., Norouzi M, Macherey W, et al.. Google's Neural Machine Translation System: Bridging the Gap between Human and Machine Translation. 2016;
43. Sang EFTK, Buchholz S. Introduction to the CoNLL-2000 Shared Task: Chunking. :127–322000;
44. Malik-Sheriff RS, Glont M, Nguyen TVN, Tiwari K, Roberts MG, Xavier A, et al.. BioModels—15 years of sharing computational models in life science. *Nucleic Acids Research*. Oxford Academic; 2020; doi: 10.1093/NAR/GKZ1055.
45. Xie Q, Luong MT, Hovy E, Le Q v.. Self-training with Noisy Student improves ImageNet classification. *Proceedings of the IEEE Computer Society Conference on Computer Vision and Pattern Recognition*. IEEE Computer Society; 2019; doi: 10.1109/CVPR42600.2020.01070.
46. Jumper J, Evans R, Pritzel A, Green T, Figurnov M, Ronneberger O, et al.. Highly accurate protein structure prediction with AlphaFold. *Nature*. Nature Publishing Group; 2021; doi: 10.1038/s41586-021-03819-2.

47. : Annotations submission service - Tools - Europe PMC. https://europepmc.org/AnnotationsSubmission Accessed 2022 Mar 14.
48. : ZOOMA. https://www.ebi.ac.uk/spot/zooma/ Accessed 2022 Mar 14.
49. : Ontology Lookup Service < EMBL-EBI. https://www.ebi.ac.uk/ols/index Accessed 2022 Mar 14.
50. Yilmaz P, Kottmann R, Field D, Knight R, Cole JR, Amaral-Zettler L, et al.. Minimum information about a marker gene sequence (MIMARKS) and minimum information about any (x) sequence (MIxS) specifications. *Nature Biotechnology 2011 29:5*. Nature Publishing Group; 2011; doi: 10.1038/nbt.1823.
51. : (ANNOTATION_PROVIDER:"Metagenomics") - Search results - Europe PMC. https://europepmc.org/search?query=%28ANNOTATION_PROVIDER%3A%22Metagenomics%22%29&page=1 Accessed 2022 Mar 14.
52. : Europe PMC - Annotations API. https://europepmc.org/AnnotationsApi Accessed 2022 Mar 14.
53. : Browse data < MGnify < EMBL-EBI. https://www.ebi.ac.uk/metagenomics/browse#publications Accessed 2022 Mar 14.
54. : Contextual Data ClearingHouse (CDCH). https://www.ebi.ac.uk/ena/clearinghouse/api/ Accessed 2022 Mar 14.
55. Yang Z, Dai Z, Yang Y, Carbonell J, Salakhutdinov R, Le Q v.. XLNet: Generalized Autoregressive Pretraining for Language Understanding. 2019;
56. Liu Y, Ott M, Goyal N, Du J, Joshi M, Chen D, et al.. RoBERTa: A Robustly Optimized BERT Pretraining Approach. 2019;
57. Clark K, Luong M-T, Le Q v., Manning CD. ELECTRA: Pre-training Text Encoders as Discriminators Rather Than Generators. *arXiv*. arXiv; 2020;
58. Callahan BJ, McMurdie PJ, Holmes SP. Exact sequence variants should replace operational taxonomic units in marker-gene data analysis. *The ISME Journal 2017 11:12*. Nature Publishing Group; 2017; doi: 10.1038/ismej.2017.119.

Figure 1

Click here to access/download;Figure;Figure 1.pdf

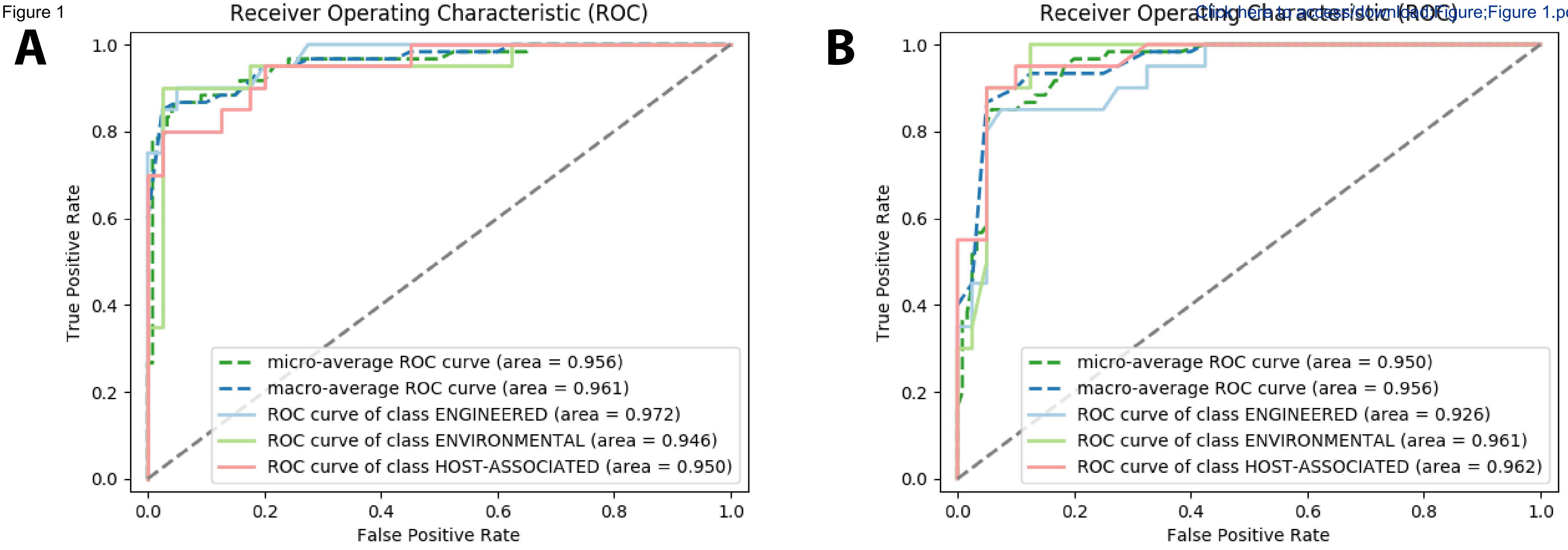

Figure 2

Click here to access/download;Figure;Figure 2.pdf

MATERIALS AND METHODS

Exposure systems.

Juvenile red snapper were obtained from, and the exposure study carried out at, the University of Southern Mississippi's Gulf Coast Research Laboratory (GCRL). Fish were exposed to the chemically enhanced water-accommodated fraction of Corexit 9500/DWH oil (CEWAF) and/or bacteria in a flowthrough system consisting of 24 tanks, each holding 75-L. Water was maintained at 20°C, 15-ppt salinity, dissolved oxygen content of >5 mg/L, and a pH of 8.5. Fish were maintained on a 16-h/8-h light/dark cycle and fed daily. Each tank held 4 fish, and each treatment was replicated in 4 tanks, for a total of 16 fish per treatment. This number was selected to allow for sampling of at least 6 individuals at two time points per treatment ($n = 12$) while providing additional individuals to account for potential mortalities. Fish were weighed in grams prior to stocking. There were six treatments, which included a control, a challenge/recovery treatment, two exposure/recovery treatments, and two chronic exposure treatments (Fig. 1): (i) Seawater Control, seawater for 28 days; (ii) Bacteria/Recovery, seawater for 7 days, a 1-h bacterial challenge, and then seawater for 21 days; (iii) CEWAF/Recovery, CEWAF for 7 days followed by clean seawater for 21 days; (iv) CEWAF/Bacteria/Recovery, CEWAF for 7 days, a 1-h pathogen challenge, and then seawater for 21 days; (v) CEWAF, CEWAF for 28 days; and (vi) CEWAF/Bacteria/CEWAF, CEWAF for 7 days, a 1-h pathogen challenge, and then CEWAF for 21 days.

During CEWAF exposure periods, fish were continuously exposed to Corexit 9500/DWH crude oil at a flow rate of 2 L/h. During periods of clean seawater exposure, fish were exposed to seawater at the same flow rate. The targeted nominal CEWAF exposure solution of 1 ppm total petroleum hydrocarbons (TPH), an environmentally relevant concentration found in Gulf of Mexico subsurface water samples collected during and after the DWH oil spill (56), was prepared fresh every 48 h following standard protocols issued by the Chemical Response to Oil Spills Ecological Effects Research Forum (CROSERF) (57). This stock CEWAF served as a source to supplement all exposure tanks at equal rates using a dilution system as in previous studies (12). Water quality (salinity, oxygen, pH, and temperature) and chemistry (ammonia, nitrite, nitrate, and alkalinity) were monitored and controlled to maintain appropriate environmental conditions during experimental trials. PAH concentrations were measured periodically in water of both exposure and control tanks, but liver PAH concentrations were used as the primary metric of PAH exposure. Bacterial challenges were performed with a *Vibrio anguillarum* strain (*Listonella anguillarum* [Bergeman] MacDonell and Colwell [ATCC 19264]), isolated from lesions in cod. This strain is of serotype O2, one of the dominant serotypes causing vibriosis in fish (25), for which Koch's postulates have been demonstrated (https://www.atcc.org/products/19264). Fish were challenged with *V. anguillarum* at a concentration of 4.0×10^5 CFU/mL of seawater. This concentration was chosen to reflect a slightly higher concentration than that used in previous research (13), as the previous study used juvenile flounder, which were smaller than the individuals used in the current study.

Measurements and morphological observations.

During the exposure experiments, fish were observed for changes in behavior and morphology. All exposures were conducted under approved protocols: Mote Marine Laboratory's Institutional Animal Care and Use Committee (IACUC; 18-04-KM2) for toxicity testing of oil and dispersed oil on marine fishes and the University of Southern

Annota
Get cita
Open PD
Claim t

exposure tanks (1) Find

Treatment

- Seawater (1) Find
- bacterial challenge (1) Find
- clean seawater (1) Find
- Corexit 9500/DWH crude oil (1) Find
- seawater (1) Find
- Vibrio anguillarum strain (1) Find
- V. anguillarum (1) Find
- dibenzothiophene-d8 (1) Find
- benzo(e)pyrene-d12 (1) Find

Kit

- DNeasy PowerSoil kit (1) Find

Primer

- 515F (5'-GTGCCAGCMGCCGCGGTAA-3') (1) Find
- 806R (5'-GGACTACHVGGGTWTCTAAT-3') (1) Find

Gene

- 18S (2) Find
- V4 (2) Find
- cDNA (1) Find
- 16S rRNA (1) Find

LCM

- Paired-end (1) Find

Sequencing

- Illumina MiSeq (1) Find
- MiSeq (1) Find

Figure 3

Click here to access/download;Figure;Figure 3.pdf

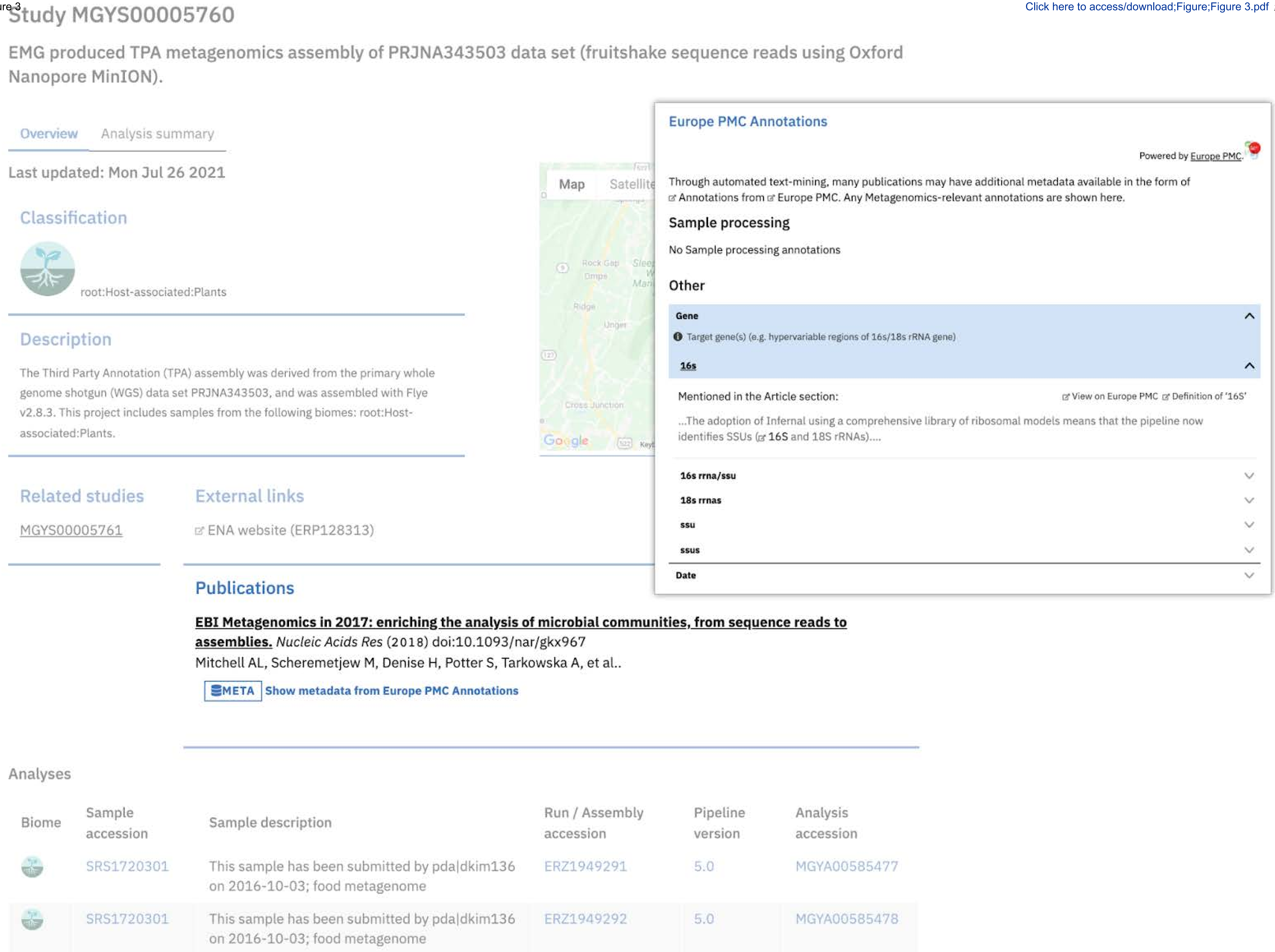

Figure 4

Click here to access/download;Figure;Figure 4.pdf

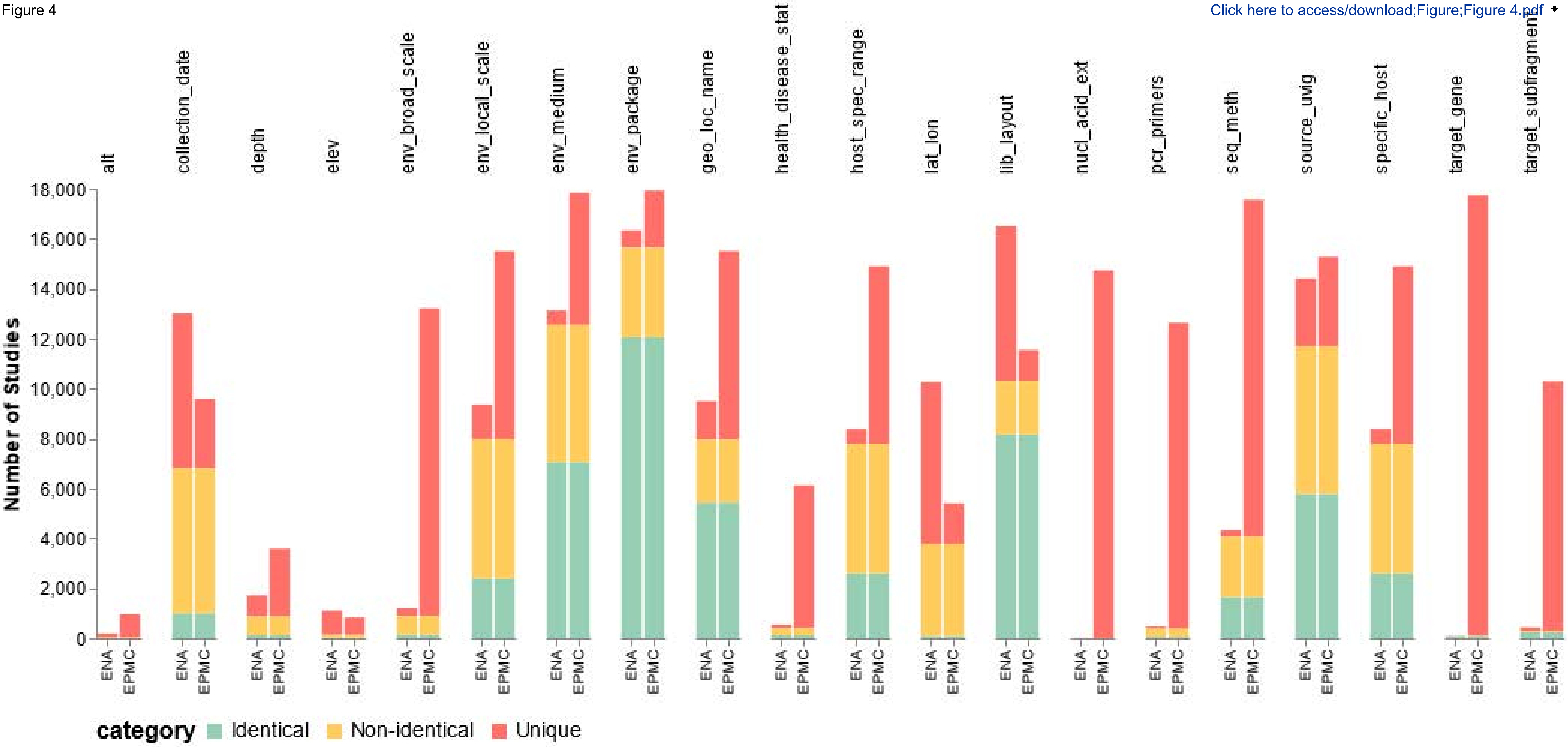

Click here to access/download
Supplementary Material
Nassar2022_supplementary.docx

Figure 4 analysis data

Click here to access/download
Supplementary Material
Figure 4 Analysis Data.csv

Figure 4 data

Click here to access/download
Supplementary Material
Figure 4 Data.csv
