# Supplementary material for: A machine learning framework for discovery and enrichment of metagenomics metadata from open access publications
Source: Gigascience. 2022 Aug 11;11:giac077. doi: 10.1093/gigascience/giac077 (PMC9366992; doi:10.1093/gigascience/giac077)
Supplement: giac077_Supplemental_File [file giac077_supplemental_file.docx]

## **A machine learning framework for discovery and enrichment of metagenomics metadata from open access publications**

Maaly Nassar, Alexander B. Rogers, Francesco Talo', Santiago Sanchez, Zunaira Shafique, Robert D. Finn, Johanna McEntyre^*^

European Molecular Biology Laboratory, European Bioinformatics Institute (EMBL-EBI), Wellcome Trust Genome Campus, Hinxton, Cambridge CB10 1SD, UK.

Corresponding. mcentyre@ebi.ac.uk

| **Hierarchical level** | **Classes** |
| --- | --- |
| **1^st^** | Engineered  Environmental  Host-associated |
| **2^nd^** | Engineered:Bioreactor  Engineered:Built environment  Engineered:Food production  Engineered:Modeled  Engineered:Wastewater  Environmental:Aquatic  Environmental:Terrestrial  Host-associated:Animal  Host-associated:Human  Host-associated:Insecta  Host-associated:Mammals  Host-associated:Plants |
| **3^rd^** | Engineered:Bioreactor:Continuous culture  Engineered:Modeled:Simulated communities (DNA mixture)  Engineered:Modeled:Simulated communities (microbial mixture)  Environmental:Aquatic:Freshwater  Environmental:Aquatic:Marine  Environmental:Aquatic:Non-marine Saline and Alkaline  Environmental:Aquatic:Thermal springs  Environmental:Terrestrial:Soil  Host-associated:Animal:Digestive system  Host-associated:Human:Digestive system  Host-associated:Human:Reproductive system  Host-associated:Human:Respiratory system  Host-associated:Human:Skin  Host-associated:Mammals:Digestive system  Host-associated:Plants:Rhizosphere |
| **4^th^** | Environmental:Aquatic:Freshwater:Groundwater  Environmental:Aquatic:Freshwater:Ice  Environmental:Aquatic:Freshwater:Lake  Environmental:Aquatic:Marine:Coastal  Environmental:Aquatic:Marine:Hydrothermal vents  Environmental:Aquatic:Marine:Intertidal zone  Environmental:Aquatic:Marine:Oceanic  Environmental:Aquatic:Marine:Sediment  Host-associated:Human:Digestive system:Large intestine  Host-associated:Human:Digestive system:Oral  Host-associated:Human:Reproductive system:Vagina  Host-associated:Mammals:Digestive system:Fecal  Host-associated:Mammals:Digestive system:Large intestine  Host-associated:Mammals:Digestive system:Stomach |
| **5^th^** | Host-associated:Human:Digestive system:Large intestine:Fecal  Host-associated:Human:Digestive system:Oral:Subgingival plaque  Host-associated:Mammals:Digestive system:Large intestine:Fecal  Host-associated:Mammals:Digestive system:Stomach:Rumen |

##### **Table 1.** Random forest GOLD multiclass hierarchical levels

| **Class** | **PMC ID** | **Prediction probability** |
| --- | --- | --- |
| Engineered | PMC5787918 | 0.6 |
|  | PMC5795077 | 0.6 |
|  | PMC6531920 | 0.6 |
|  | PMC4475827 | 0.5 |
|  | PMC5603099 | 0.5 |
|  | PMC6328780 | 0.5 |
|  | PMC6133121 | 0.5 |
|  | PMC4844624 | 0.5 |
|  | PMC4295556 | 0.5 |
|  | PMC5219019 | 0.5 |
|  | PMC6614595 | 0.5 |
|  | PMC6204281 | 0.5 |
|  | PMC6424877 | 0.5 |
|  | PMC5583138 | 0.5 |
|  | PMC5486833 | 0.5 |
|  | PMC5748143 | 0.5 |
|  | PMC6068774 | 0.5 |
|  | PMC5716999 | 0.5 |
|  | PMC6011948 | 0.5 |
|  | PMC4912747 | 0.5 |
|  | PMC4554467 | 0.5 |
|  | PMC6247018 | 0.5 |
|  | PMC6611852 | 0.5 |
|  | PMC6172326 | 0.5 |
|  | PMC5450463 | 0.5 |
|  | PMC4970986 | 0.5 |
|  | PMC6375858 | 0.5 |
|  | PMC5099235 | 0.5 |
|  | PMC4493836 | 0.5 |
|  | PMC6470552 | 0.4 |
|  | PMC5049776 | 0.4 |
|  | PMC4585762 | 0.4 |
|  | PMC5758579 | 0.4 |
|  | PMC5599880 | 0.4 |
|  | PMC5460018 | 0.4 |
|  | PMC5831590 | 0.4 |
|  | PMC5910564 | 0.4 |
|  | PMC5942161 | 0.4 |
|  | PMC5080403 | 0.4 |
|  | PMC6236004 | 0.4 |
|  | PMC4703780 | 0.4 |
|  | PMC6390037 | 0.4 |
|  | PMC6446978 | 0.4 |
|  | PMC5481317 | 0.4 |
| Environmental | PMC5864207 | 0.6 |
|  | PMC5554446 | 0.6 |
|  | PMC4623418 | 0.6 |
|  | PMC6014231 | 0.6 |
|  | PMC5498550 | 0.6 |
|  | PMC5035745 | 0.6 |
|  | PMC6315191 | 0.6 |
|  | PMC6598672 | 0.6 |
|  | PMC6305388 | 0.6 |
|  | PMC6486251 | 0.6 |
|  | PMC6813490 | 0.5 |
|  | PMC5808123 | 0.5 |
|  | PMC4023046 | 0.5 |
|  | PMC5520028 | 0.5 |
|  | PMC5209421 | 0.5 |
|  | PMC6002407 | 0.5 |
|  | PMC4703759 | 0.5 |
|  | PMC5575242 | 0.5 |
|  | PMC5812542 | 0.5 |
|  | PMC5474096 | 0.5 |
|  | PMC6673769 | 0.5 |
|  | PMC6310837 | 0.5 |
|  | PMC5943533 | 0.5 |
|  | PMC6776777 | 0.5 |
|  | PMC4814466 | 0.5 |
|  | PMC6225844 | 0.5 |
|  | PMC5797777 | 0.5 |
|  | PMC5492102 | 0.5 |
|  | PMC5827536 | 0.5 |
|  | PMC6759336 | 0.5 |
|  | PMC4852812 | 0.5 |
|  | PMC4556746 | 0.5 |
|  | PMC6052102 | 0.5 |
|  | PMC6616603 | 0.5 |
|  | PMC5994334 | 0.5 |
|  | PMC6298960 | 0.5 |
|  | PMC6137086 | 0.5 |
|  | PMC5069773 | 0.5 |
|  | PMC6071827 | 0.5 |
|  | PMC4724708 | 0.5 |
|  | PMC4628106 | 0.5 |
|  | PMC5742206 | 0.5 |
|  | PMC5852596 | 0.5 |
|  | PMC5568565 | 0.4 |
|  | PMC6381156 | 0.4 |
|  | PMC5440473 | 0.4 |
|  | PMC5552935 | 0.4 |
|  | PMC4564720 | 0.4 |
|  | PMC5423328 | 0.4 |
|  | PMC6249378 | 0.4 |
| Host-associated | PMC5320696 | 0.7 |
|  | PMC6159892 | 0.7 |
|  | PMC6435815 | 0.7 |
|  | PMC5268494 | 0.6 |
|  | PMC5705695 | 0.6 |
|  | PMC6498444 | 0.6 |
|  | PMC5887201 | 0.6 |
|  | PMC6033232 | 0.6 |
|  | PMC6777474 | 0.6 |
|  | PMC5933315 | 0.6 |
|  | PMC5333848 | 0.6 |
|  | PMC4950857 | 0.6 |
|  | PMC6462939 | 0.6 |
|  | PMC6538789 | 0.6 |
|  | PMC4544774 | 0.6 |
|  | PMC5938040 | 0.6 |
|  | PMC5765694 | 0.6 |
|  | PMC5021991 | 0.6 |
|  | PMC6019644 | 0.6 |
|  | PMC4669145 | 0.6 |
|  | PMC4113139 | 0.6 |
|  | PMC6767489 | 0.6 |
|  | PMC5766553 | 0.6 |
|  | PMC5967468 | 0.5 |
|  | PMC4106219 | 0.5 |
|  | PMC6475661 | 0.5 |
|  | PMC6302507 | 0.5 |
|  | PMC6258300 | 0.5 |
|  | PMC5852056 | 0.5 |
|  | PMC6594611 | 0.5 |
|  | PMC5592222 | 0.5 |
|  | PMC5923270 | 0.5 |
|  | PMC4881475 | 0.5 |
|  | PMC6544264 | 0.5 |
|  | PMC6321908 | 0.5 |
|  | PMC5702186 | 0.5 |
|  | PMC5935533 | 0.5 |
|  | PMC5997989 | 0.5 |
|  | PMC5524138 | 0.5 |
|  | PMC4767880 | 0.5 |
|  | PMC5371668 | 0.5 |
|  | PMC4636265 | 0.5 |
|  | PMC6402632 | 0.4 |
|  | PMC6034077 | 0.4 |
|  | PMC4834568 | 0.4 |
|  | PMC5322875 | 0.4 |

##### **Table 2.** Literature triage classified into 3 biome classes, using TF-IDF random forest model

| **Section** | **Class** | **Precision** | **Recall** | **F1-Score** | **Support** |
| --- | --- | --- | --- | --- | --- |
| **Abstract** | Engineered | 0.58 | 0.75 | 0.65 | 20 |
|  | Environmental | 0.78 | 0.7 | 0.74 | 20 |
|  | Host-associated | 0.81 | 0.65 | 0.72 | 20 |
|  | **macro-average** | **0.72** | **0.70** | **0.70** | **60** |
| **Introduction** | Engineered | 0.58 | 0.7 | 0.64 | 20 |
|  | Environmental | 0.94 | 0.75 | 0.83 | 20 |
|  | Host-associated | 0.75 | 0.75 | 0.75 | 20 |
|  | **macro-average** | **0.76** | **0.73** | **0.74** | **60** |
| **Method** | Engineered | 0.77 | 0.85 | 0.81 | 20 |
|  | Environmental | 0.88 | 0.75 | 0.81 | 20 |
|  | Host-associated | 0.9 | 0.95 | 0.93 | 20 |
|  | **macro-average** | **0.85*** | **0.85*** | **0.85*** | **60** |
| **Results** | Engineered | 0.7 | 0.88 | 0.78 | 16 |
|  | Environmental | 0.88 | 0.88 | 0.88 | 16 |
|  | Host-associated | 0.92 | 0.69 | 0.79 | 16 |
|  | **macro-average** | **0.83** | **0.81** | **0.81** | **48** |
| **Discussion** | Engineered | 0.67 | 0.67 | 0.67 | 18 |
|  | Environmental | 0.74 | 0.78 | 0.76 | 18 |
|  | Host-associated | 0.88 | 0.83 | 0.86 | 18 |
|  | **macro-average** | **0.76** | **0.76** | **0.76** | **54** |

##### **Table 3.** The performance metrics of TF-IDF biome classifiers when trained on abstract, introduction, method or discussion texts only. *classifiers trained on method sections showed the highest precision, recall and F1-score macro-averages.

| **Database** | **Query** |
| --- | --- |
| **ENA** | {'query':'tax_tree(408169) OR environmental_sample=true OR library_source="METATRANSCRIPTOMIC" OR library_source="METAGENOMIC"','result':'read_study'} |
| **BioProject** | (txid408169[Organism:exp] OR metagenome OR metagenomic OR microbiota OR microbiome OR metagenomics OR ((microbial OR bacterial) AND (community OR communities OR population OR populations))) |
| **Europe PMC** | ((KW:"metagenomics" OR (KW:"gastrointestinal" AND KW:"microbiome") OR KW:"microbiota" OR KW:"mycobiome" OR KW:"metagenome" OR (KW:"microbiology" AND (KW:"environmental" OR (KW:"geologic" OR KW:"sediments") OR (KW:"waste" OR KW:"water") OR KW:"groundwater" OR KW:"ecosystem" OR KW:"air" OR KW:"rivers" OR (KW:"ice" AND KW:"cover") OR KW:"soil" OR KW:"petroleum" OR (KW:"bioelectric" OR (KW:"energy" AND KW:"source")) OR (KW:"biodegradation") OR KW:"caves" OR KW:"food" OR KW:"industrial" OR (KW:"protective" AND KW:"clothing") OR KW:"biofuels" OR KW:"plants" OR KW:"insecta" OR KW:"parasites" OR KW:"animal" OR KW:"human")) OR (KW:"metagenome" AND KW:"genetics") OR (KW:"microbiology" AND (TITLE:"biosynthetic gene cluster" OR ABSTRACT:"biosynthetic gene cluster" OR TITLE:"secondary metabolite" OR ABSTRACT:"secondary metabolite" OR KW:"biological products")) OR TITLE:"microbiome" OR ABSTRACT:"microbiome" OR TITLE:"microbiota" OR ABSTRACT:"microbiota" OR TITLE:"biome" OR ABSTRACT:"biome" OR TITLE:"virome" OR ABSTRACT:"virome" OR TITLE:"mycobiome" OR ABSTRACT:"mycobiome" OR TITLE:"microbial fuel cells" OR ABSTRACT:"microbial fuel cells" OR TITLE:"metagenome" OR ABSTRACT:"metagenome" OR TITLE:"metagenomes" OR ABSTRACT:"metagenomes" OR TITLE:"metagenomic" OR ABSTRACT:"metagenomic" OR TITLE:"metagenomics" OR ABSTRACT:"metagenomics" OR TITLE:"biodegradation" OR ABSTRACT:"biodegradation") AND (SRC:MED OR SRC:PMC OR SRC:AGR OR SRC:CBA OR PUB_TYPE:REVIEW)) |
| **PMC** | txid408169[Organism:exp] |
| **PubMed** | ("Metagenomics"[MH] OR "Gastrointestinal Microbiome"[MH] OR "Microbiota"[MH] OR "Mycobiome"[MH] OR "Metagenome"[MH] OR "Environmental/microbiology"[MH] OR "Geologic Sediments/microbiology"[MH] OR "Waste Water/microbiology"[MH] OR "Groundwater/microbiology"[MH] OR "Ecosystem/microbiology"[MH] OR "Air Microbiology"[MH] OR "Water Microbiology"[MH] OR "Rivers/microbiology"[MH] OR "Ice Cover/microbiology"[MH] OR "Soil Microbiology"[MH] OR "Petroleum/microbiology"[MH] OR "Bioelectric Energy Sources/microbiology"[MH] OR "Biodegradation, Environmental"[MH] OR "Environmental Microbiology"[MH] OR "Caves/microbiology"[MH] OR "Food Microbiology"[MH] OR "Industrial Microbiology"[MH] OR "Protective Clothing/microbiology"[MH] OR "Biofuels/microbiology"[MH] OR "Plants/microbiology"[MH] OR "Insecta/microbiology"[MH] OR "Parasites/microbiology"[MH] OR "microbiome" [TIAB] OR "microbiota" [TIAB] OR "biome"[TIAB] OR "virome"[TIAB] OR "mycobiome"[TIAB] OR "microbial fuel cells"[TIAB] OR "metagenome"[TIAB] OR "metagenomes"[TIAB] OR "metagenomics"[TIAB] OR "metagenomic"[TIAB] OR "Metagenome/genetics"[MH] OR ("microbiology"[MH] AND ("biosynthetic gene cluster"[TIAB] OR "secondary metabolite"[TIAB] OR "biological products"[MH]))) |

##### **Table 4.** Queries used in retrieving metagenomics studies and publications from ENA, BioProject, Europe PMC, PMC and PubMed.

| **Classifier features** | **Class** | **Precision** | **Recall** | **F1-Score** | **Support** |
| --- | --- | --- | --- | --- | --- |
| **TF-IDF** | Abstract | 0.82 | 0.97 | 0.89* | 1000 |
|  | Introduction | 0.93 | 0.93 | 0.93* | 1000 |
|  | Methods | 0.93 | 0.9 | 0.91* | 1000 |
|  | Results | 0.91 | 0.87 | 0.89* | 1000 |
|  | Discussion | 0.93 | 0.83 | 0.88* | 1000 |
| **Doc2Vec^ENA^** | Abstract | 0.78 | 0.87 | 0.82 | 1000 |
|  | Introduction | 0.75 | 0.83 | 0.79 | 1000 |
|  | Methods | 0.73 | 0.6 | 0.66 | 1000 |
|  | Results | 0.69 | 0.62 | 0.65 | 1000 |
|  | Discussion | 0.64 | 0.68 | 0.66 | 1000 |

##### **Table 5.** The performance metrics of abstract, introduction, method, results and discussion sections classifiers. Classifiers features are either TF-IDF or Doc2Vec**^ENA^** (embeddings generated from training ENA cross-referenced publications). Support is the number of publications in test datasets per class.


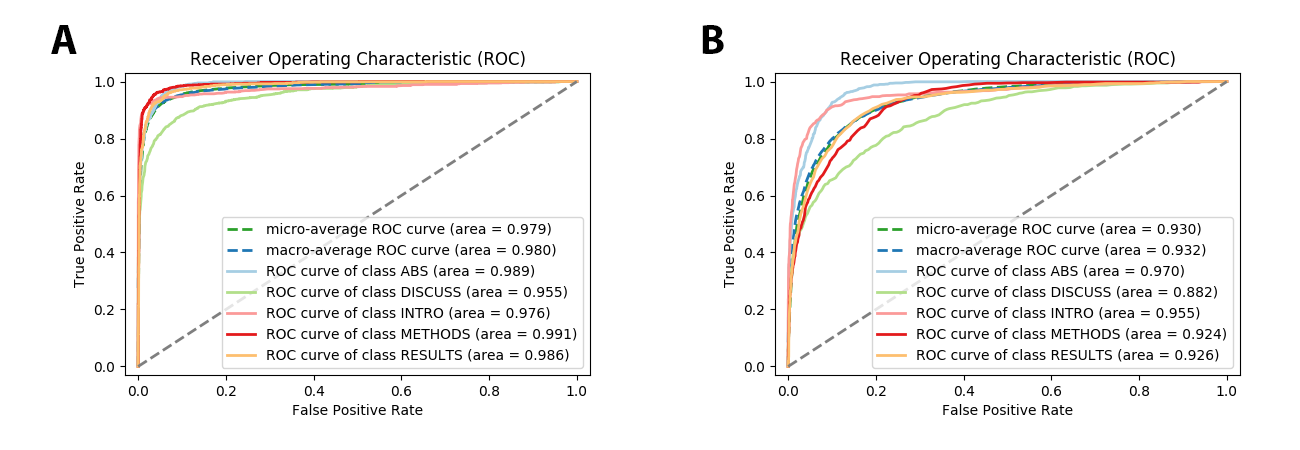


##### **Figure 1.** Receiver operating characteristic (ROC) curves of abstract (ABS), introduction(INTRO), methods (METHODS), results (RESULTS) and discussion (DISCUSS) sections classifiers, using TF-IDF (A) or Doc2Vec**^ENA^** (B) as training features.

| **Entity** | **Learning Rate** | **Epoch** | **Recall** | **Precision** | **F1-Score** |
| --- | --- | --- | --- | --- | --- |
| **Ecoregion** | 4e-5 | 30 | 0.74 | 0.85 | 0.78 |
| **Host** | 1e-5 | 30 | 0.89 | 0.76 | 0.82 |
| **Engineered** | 2e-5 | 10 | 0.69 | 0.83 | 0.75 |
| **Date** | 4e-5 | 10 | 0.83 | 0.87 | 0.85 |
| **Place** | 5e-5 | 10 | 0.88 | 0.87 | 0.88 |
| **Site** | 3e-5 | 30 | 0.69 | 0.78 | 0.73 |
| **Body-Site** | 3e-5 | 10 | 0.91 | 0.88 | 0.9 |
| **Sample-Material** | 2e-5 | 10 | 0.75 | 0.82 | 0.78 |
| **State** | 5e-5 | 30 | 0.76 | 0.8 | 0.78 |
| **Treatment** | 1e-5 | 10 | 0.74 | 0.8 | 0.77 |
| **Kit** | 5e-5 | 30 | 0.96 | 0.93 | 0.95 |
| **Primer** | 2e-5 | 10 | 0.9 | 0.92 | 0.91 |
| **Gene** | 3e-5 | 10 | 0.93 | 0.9 | 0.92 |
| **LS** | 2e-5 | 10 | 0.92 | 0.88 | 0.89 |
| **LCM** | 4e-5 | 30 | 0.96 | 0.99 | 0.97 |
| **Sequencing** | 2e-5 | 10 | 0.94 | 0.89 | 0.92 |

##### **Table 6:** Token-wise precision, recall and F1-score macro-averages of the 16-best performance NER models trained on predicted datasets.

| **MIxS Field** | **ENA Field** | **Metagenomics Entity** |
| --- | --- | --- |
| **lat_lon** | geographic location (latitude), geographic location (longitude), lat lon, lat_lon, latitude, longitude | Place |
| **depth** | Depth, depth, geographic location (depth) | Site |
| **alt** | alt, altitude | Site |
| **elev** | elev, elevation, geographic location (elevation) | Site |
| **geo_loc_name** | country, geo_loc_name, geographic location (country and/or sea), geographic location (country and/or sea,region), geographic location (region and locality) | Place |
| **collection_date** | collection date, collection_date, collection_timestamp | Date |
| **env_broad_scale** | ecotype, env_broad_scale | Ecoregion, Engineered |
| **env_local_scale** | body_habitat, body_site, env_local_scale, host body site, host_body_habitat, host_body_site, host_tissue_sampled, isolation source, isolation-source, isolation_source, source_name, tissue, tissue_type | Site, Body-Site |
| **env_medium** | body_product, env_material, env_matter, env_medium, environment (material), host_body_product, host_tissue_sampled, isolation source, isolation-source, isolation_source, material, ref_biomaterial, sample_type, sediment environmental package, soil environmental package, source_name, tissue, tissue_type, water environmental package | Sample-Material |
| **specific_host** | common_name, host, host common name, host scientific name, host_common_name, specific host, specific_host | Host |
| **host_spec_range** | common_name, host, host common name, host scientific name, host_common_name, specific host, specific_host | Host |
| **health_disease_stat** | disease, health_disease_stat, health_state, host health state, host_disease | state |
| **source_uvig** | investigation type, investigation_type, library_strategy, source_uvig | LS |
| **lib_layout** | lib_layout, library_layout | LCM |
| **target_gene** | target gene, target_gene, tax_ident | Gene |
| **target_subfragment** | target subfragment, target_subfragment | Gene |
| **pcr_primers** | pcr primers, pcr_primers, primer | Primer |
| **seq_meth** | illumina_technology, instrument_model, platform, seq_meth, seq_methods, sequencing method, sequencing_meth | Sequencing |
| **nucl_acid_ext** | nucl_acid_ext | Kit |
| **env_package** | Age, Sex, Treatment, age, ammonium, biome, body_habitat, body_product, body_site, breed, chem_administration, dev_stage, diet, disease, env_biome, env_feature, env_material, env_matter, env_package, environment (biome), environment (feature), environment (material), environmental package, ethnicity, feature, gender, health_state, host age, host body site, host common name, host health state, host scientific name, host sex, host-associated environmental package, host_age, host_body_habitat, host_body_mass_index, host_body_product, host_body_site, host_diet, host_disease, host_genotype, host_height, host_life_stage, host_phenotype, host_sex, host_tissue_sampled, human gut environmental package, human-associated environmental package, isolation source, isolation-source, isolation_source, life_stage, nitrate, organism, pH, perturbation, ph, phosphate, plant-associated environmental package, ref_biomaterial, salinity, sample_type, scientific_name, sediment environmental package, sex, soil environmental package, specific host, specific_host, temp, temperature, tissue, tissue_type, tot_nitro, treatment, water environmental package | Host, Ecoregion, Engineered, State, Treatment, Site, Body-Site |

##### **Table 7:** Mapping ENA metadata fields and metagenomics entities to MIxS checklist

| **Study** | **PMC** | **MIxS** | **ENA Metadata** | **Metagenomics Annotations** |
| --- | --- | --- | --- | --- |
| PRJDB3293 | PMC7105989 | specific_host | ['Potamochoerus porcus', '**Sus scrofa** domesticus', '**Sus scrofa** scrofa'] | [**'Sus scrofa'**, 'boars: tetQ', 'pigs', 'wild boar', 'wild boars'] |
| PRJDB8953 | PMC7333650 | lat_lon | [**'34.333958 N 136.692204 E'**, '34.44085 N 136.31307 E'] | [**'34.333958 N 136.692204 E'**] |
| PRJEB11763 | PMC4700446, PMC5684312 | seq_meth | [**'454 GS FLX'**, 'ls454',  'pyrosequencing'] | [**'454 GS FLX**+ pyrosequencer'] |
| PRJDB10210 | PMC8407723 | source_uvig | ['**AMPLICON**'] | ['**amplicon**'] |
| PRJDB9252 | PMC8121002, PMC7409863 | collection_date | ['2018-07-20'] | ['20 July 2018'] |
| PRJDB9200 | PMC8534475 | depth | ['982 m'] | ['982-m depth'] |
| PRJDB5495 | PMC8322762 | env_medium | ['**skin** from ulcerative lesion of bovine foot infected with digital dermatitis'] | ['DD lesion skin', 'Skin samples', 'biopsy punches', **'skin', 'skin** lesion samples'] |
| PRJDB5860 | PMC6024022 | specific_host | ['Homo sapiens'] | ['Subjects', 'children', 'parents/guardians', 'participants', 'subjects'] |
| PRJDB6715 | PMC6307992 | depth | ['1150 m', '1154 m', '1385 m', '1426 m', '1432 m', '1483 m', '1839 m', '2748 m', '2792 m', '2887 m', '2888 m', '2986 m', '3198 m', '3199 m', '3200 m', '4287 m', '4290 m', '4374 m', '4479 m', '4480 m', '5373 m', '5507 m', '5517 m', '5577 m', '5582 m'] | ['water depths of 1,150–5,520 m'] |
| PRJEB11804 | PMC4785176 | geo_loc_name | ['United Kingdom'] | ['52°42′N, 4°09′W', 'Aberystwyth beach', 'UK'] |
| PRJEB11804 | PMC4785176 | seq_meth | ['Ion Torrent PGM', 'ion_torrent',  'PGM Ion-Torrent'] | ['Ion PGM', 'Ion Torrent Personal Genome Machine (PGM) system', 'pyrosequencing'] |
| PRJDB1602 | PMC4567559 | source_uvig | ['WGS'] | ['TruSeq', 'metagenomics', 'shotgun'] |
| PRJDA73021 | PMC3218302 | geo_loc_name | - | ['51°22.8′N, 11°43.1′W', 'Japan', 'Porcupine Seabight', 'Site U1317'] |
| PRJDB8477 | PMC7734398 | pcr_primers | - | ['341F (5′-TCGTCGGCAGCGTCAGATGTGTATAAGAGACAGCCTACGGGNGGCWGCAG-3′)'] |
| PRJDB9293 | PMC8147061 | target_gene | - | ['16S rRNA', 'V3-V4'] |
| PRJDB5614 | PMC5745017 | nucl_acid_ext | - | ['RNA PowerSoil total RNA isolation kit', 'RNeasy', 'RNeasy PowerSoil total RNA kit', 'RNeasy mini kit'] |
| PRJDB7448 | PMC7224477 | elev | - | ['5 m above the sea floor'] |
| PRJDB7448 | PMC7224477 | env_local_scale | - | ['0–1,000 m', '0–1,000 m depth', '0–200 m', '10 m depth', '200 m', '5 m above the sea floor', 'arctic', 'depth', 'depth < 200 m in', 'epipelagic', 'epipelagic layer', 'lower mesopelagic', 'mesopelagic', 'mesopelagic (200–500 m', 'mouth-opening', 'sampling', 'subarctic region', 'upper mesopelagic', 'upper-layer', 'water surface'] |
| PRJEB11827 | PMC4971113 | health_disease_stat | - | ['Italian healthy', 'None of the participants had any history of GI abnormalities'] |
| PRJEB11799 | PMC7359306 | lat_lon | ['46.49587773', '46.49633659', '9.931472421', '9.932285399'] | - |
| PRJDB10112 | PMC7686419 | collection_date | ['2018-11-27'] | - |
| PRJDB6461 | PMC7936642 | lib_layout | ['single'] | ['paired reads', 'paired-end'] |
| PRJDB10611 | PMC7966939 | collection_date | ['2020-05-17', '2020-05-23', '2020-05-31', '2020-06-20', '2020-07-02', '2020-07-13'] | ['June 21th, 2003', 'May 15th, 2003'] |
| PRJDB5850 | PMC5809899 | env_medium | ['anode'] | ['activated sludge', 'wastewater'] |
| PRJDB5860 | PMC6024022 | seq_meth | ['454 GS FLX Titanium' , 'ls454'  ] | ['Illumina MiSeq'] |
| PRJDB5936 | PMC6005635 | depth | ['0.0 m'] | ['deeper than ~30 cm from the river surface'] |

##### **Table 8:** Examples from matching ENA metadata with their metagenomics annotations counterparts. Cases for exact matches highlighted in bold (PRJDB3293, PRJDB8953, PRJEB11763, PRJDB10210), mismatches due inconsistent data synonyms or formats (PRJDB9252, PRJDB9200, PRJDB5495, PRJDB5860, PRJDB6715, PRJEB11804, PRJDB1602), missing ENA metadata (PRJDA73021, PRJDB8477, PRJDB9293, PRJDB5614, PRJDB7448, PRJDB8518, PRJEB11766, PRJEB11827), missing metagenomics annotations (PRJEB11799, PRJDB10112) and incorrect/inaccurate ENA metadata (PRJDB6461, PRJDB10611, PRJDB5850, PRJDB5860, PRJDB5936).

| **Entity** | **Curators Annotations** | **Models Predictions** |
| --- | --- | --- |
| **Site** | For comparison, we also collected one barite-rich silica chimney (SiCh) from the extinct vent area (Figure 1G) at 7333.99N and 0809.58E at a water depth of 2367 m. An overview of examined mat-samples and chimney sub-samples, respectively, are given in Table 1. | For comparison, we also collected one barite-rich silica chimney (SiCh) from the extinct vent area (Figure 1G) at 7333.99N and 0809.58E at a water depth of 2367 m. An overview of examined mat-samples and chimney sub-samples, respectively, are given in Table 1. |
| **State** | Blood (serum lipid) analysis For triglycerides, cholesterol, LDL, and HDL levels, blood samples were taken prior to the start of the synbiotic supplementation and at and at one-month post-supplementation and submitted for serum lipid profile analysis. | Blood (serum lipid) analysis For triglycerides, cholesterol, LDL, and HDL levels, blood samples were taken prior to the start of the synbiotic supplementation and at one-month post-supplementation and submitted for serum lipid profile analysis. |
| **Treatment** | For K/BxN colonization experiments, three-week-old SPF K/BxN mice were treated with ampicillin (1g/L), neomycin (1g/L), and metronidazole (1g/L) for 10 days. | For K/BxN colonization experiments, three-week-old SPF K/BxN mice were treated with ampicillin (1g/L), neomycin (1g/L), and metronidazole (1g/L) for 10 days. |
| **Engineered** | Furthermore, at this experimental stage, batch systems were utilized to avoid potential heterogeneity of granular sludge resulting from plug-flow hydraulic regimes associated with the operation of sludge bed up-flow reactors (Wu and Hickey, 1997). | Furthermore, at this experimental stage, batch systems were utilized to avoid potential heterogeneity of granular sludge resulting from plug-flow hydraulic regimes associated with the operation of sludge bed up-flow reactors (Wu and Hickey, 1997). |

##### **Table 9:** Examples from test datasets showing entities models predictions versus curators annotations.

##### **Curation guidelines**

###### **Objectives**

Identify and annotate any term pertinent to metagenomics sample and experimental methods in metagenomics publications (literature triage).

###### **Literature Triage**

A total of 140 manuscripts covering ecoregion, host-associated and engineered microbiome environments were randomly selected for curators to annotate in 6 batches.

###### **Web Application**

Curators were invited to register and use hypothes.is (https://web.hypothes.is/). After registering, they were able to curate the manuscripts and post their annotations to EMERALD metagenomics hypothesis group.

###### **Curators Tasks**

Method section was the main section to be annotated in manuscripts. Curators' tasks were to annotate any term describing the following entities:

1. **Sample-Material:** biosample material
2. **Ecoregion:** biosample ecoregion
3. **Engineered:** biosample man-made environment
4. **Host:** biosample host
5. **Date:** biosample collection date
6. **Place:** biosample geographical place
7. **Body-Site:** biosample body site
8. **Site:** biosample site
9. **State:** state of host or environment
10. **Treatment:** treatment applied on host or environment
11. **Kit:** nucleic acid extraction kit
12. **Primer:** PCR primer
13. **Gene:** target gene or hypervariable regions
14. **LS:** library source or strategy
15. **LCM:** library layout or construction method
16. **Sequencing:** sequencing platform

###### **Metagenomics Entities**

1. **Sample-Material**
   1. **Terms to be annotated:** Any term that represents the material of the sample (e.g. ice, metal, loam, cryoconite, grass, groundwater, freshwater, seawater, wastewater, storm water, coalbed water, mine water, mine drainage, cave water, pulp and paper waste water, soil, sand, clay, composite, sediment, sediment, garden dump, silt, slime, solid waste, sludge, saliva, cerebrospinal fluid, feces, milk, blood, swab, subgingival plaque, supragingival plaque, biofilm, air, crop, fermented bevereges, dairy products, fermented vegetables, fermented seafood, activated sludge, drainage pipe biofilm, marine sediment inoculum )
   2. **Annotation tags:** Sample-Material
   3. **Examples:**
      1. “Samples in this study originate from nasopharyngeal aspirates, serum, feces and cerebrospinal fluid (csf).” ([PMC5758519](https://europepmc.org/article/MED/29311716)).
      2. “The cryoconite sample was collected near the glacier base at 2,700 m above sea level.” ([PMC5471330](https://europepmc.org/article/MED/28663747))
2. **Ecoregion**
   1. **Terms to be annotated:** The names of the ecoregion, where the sample came from (e.g. lake, pond, desert, grasslands, shrubland, forest, mangrove, swamp, rainforest, springs, river, sea, ocean).
   2. **Annotation tags:** Ecoregion
   3. **Examples:**
      1. “Once in the ocean, plastics are rapidly colonized by complex microbial communities.” ([PMC6550384](https://europepmc.org/article/MED/31166981)).
      2. “Soils cores were collected from three sites representing a thaw gradient of minimally, moderately and extensively thawed conditions within the discontinuous permafrost zone from the Eight Mile Lake (EML) watershed, west of Healy, Alaska, USA (63°52′42.1′′N, 149°15′12′′W, 700 m.a.s.l).” ([PMC5121533](https://europepmc.org/article/MED/27933054))
3. **Engineered**
   1. **Terms to be annotated:** Any term that represents a man-made environment from which the sample came from (e.g. bioreactor, farm, subway, space station, building, food, microbial fuels cells).
   2. **Annotation tags:** Engineered
   3. **Examples:**
      1. “Sewage influent samples were collected from 71 cities and 78 wastewater treatment plant (WWTP) sites from across the United States during August 2012 (7 August 2012 to 7 September 2012), January 2013 (9 January 2013 to 28 February 2013), and May 2013 (28 April 2013 to 4 June 2013).”([PMC4358014](https://europepmc.org/article/MED/25714718))
      2. “Sampling of Hungarian broilers was performed on freshly slaughtered animals at two slaughter houses under the supervision and with the permission of the district veterinary officer and poultry owners. BN can be contacted for further information on Hungarian samples.” ([PMC4199679](https://europepmc.org/article/MED/25329397))
      3. “Indoor and outdoor air samples were collected at five daycare centers (sites A to E) and five elementary schools (sites F to J) located in Seoul, Korea from August 19 to October 24, 2013.” ([PMC4447338](https://europepmc.org/article/MED/26020512))
4. **Host**
   1. **Terms to be annotated:** The names (English or Latin names) of the host, where the sample came from (e.g. human, mammals, fungi, fish, fossil, birds, mollusca, amphibia, dinoflagellates, echinodermata, porifera, plants, algae, arthropoda, coral, reptiles, sea_urchin, insecta, ectosymbionts, protozoa, epiphytes, endosymbionts)
   2. **Annotation tags:** Host
   3. **Examples:**
      1. “The 77 Armadillidium vulgare used in this study were sampled from four laboratory lineages and two field sites in France (Table 1) and were partly the same as those used in previous studies” ([PMC5934373](https://europepmc.org/article/MED/29725059))
      2. “Sampling the same shark twice was avoided by taking pictures of each side of the first dorsal fin to document individual markings on each shark, an approach which is commonly used for identification of individuals.” ([PMC7807711](https://europepmc.org/article/MED/33499949)).
5. **Date**
   1. **Terms to be annotated:** Any term that represents the collection date of the sample
   2. **Annotation tags:** Date
   3. **Examples:**
      1. “Indoor and outdoor air samples were collected at five daycare centers (sites A to E) and five elementary schools (sites F to J) located in Seoul, Korea from August 19 to October 24, 2013.” ([PMC4447338](https://europepmc.org/article/MED/26020512))
      2. “Sampling was performed in September 2006 on Jamtalferner (47.51°N, 10.09°E), a medium sized valley glacier at the southern margin of Jam valley in the Silvretta group near the “Drei-Ländereck” in Austria. It covers an area of 3.5 km2 over a length of about 2.4 km. The lowest part of the glacier is located at an altitude of 2,420 m, the highest at Hintere Jamspitze (3,156 m). The cryoconite sample was collected near the glacier base at 2,700 m above sea level.” ([PMC5471330](https://europepmc.org/article/MED/28663747))
6. **Place**
   1. **Terms to be annotated:** Any term that represents the geographical location of the sample.
   2. **Annotation tags:** Place
   3. **Examples:**
      1. “Hypolithic samples were collected from the Namib Desert (S 23°32.031′, E 15°01.813′: April 2010) (Vikram et al. 2016) and in Antarctica (78°60′, 164°00′E: January 2012) as described elsewhere (Makhalanyane, Valverde, Birkeland, et al. 2013; Makhalanyane, Valverde, Lacap, et al. 2013).” ([PMC5630931](https://europepmc.org/article/MED/27503299))
      2. “Sampling was performed in September 2006 on Jamtalferner (47.51°N, 10.09°E), a medium sized valley glacier at the southern margin of Jam valley in the Silvretta group near the “Drei-Ländereck” in Austria.” ([PMC5471330](https://europepmc.org/article/MED/28663747))
7. **Body-Site**
   1. **Terms to be annotated:** Any term that represents the organ or the tissue of the sample (e.g.intestine, throat, introitus, gut, lung, lymph nodes,kidneys, lumen, dudenal, trachea, glands, gills, rectum, rhizome, root, rumen, skin, stomach, shell, nasopharyngeal, naris, cuticle, oral cavity, umbilicus, cecum, bone, brain, axilla, p3 segment, p1 segment, posterior fornix, reproductive system, respiratory system, anterior nares, hard palate, large intestine, small intestine, digestive system, digestive tube, periodontal pockets, gastrointestinal tract, egg capsule, nervous system, integument, thoracic segment, palatine tonsils, tongue dorsum, endoperiotrophic space, sigmoid colon, pulmonary system, midpoint vagina, retroauricular crease, protodeal segment, gingiva, nasal cavity, lymphatic system, buccal mucosa, excretory system, medial distal leg, subcuticular space)
   2. **Annotation tags:** Body-Site
   3. **Examples:**
      1. “Female salivary glands are also collected and analysed.” ([PMC6172810](https://europepmc.org/article/MED/30286722))
      2. “Samples in this study originate from nasopharyngeal aspirates, serum, feces and cerebrospinal fluid (csf).” ([PMC5758519](https://europepmc.org/article/MED/29311716))
      3. “Seven anatomical locations were collected using DNA-Free, sterile cotton-tipped applicators: the external auditory canal, eyes, nose, mouth, umbilicus, rectum, and trabecular space of the occipital bone.” ([PMC6417727](https://europepmc.org/article/MED/30870464))
8. **Site**
   1. **Terms to be annotated:** Any term that represents the site of the sample within ecoregion or engineered environments (e.g. depth, altitude, hypolimnion, epilmnion, landfill, estuary, fumaroles, rhizosphere, swamp, pelagic, coastal, benthic, beach, bog, marsh, deep subsurface, oil field, oil seeps, oil reservoir, mid-stream, land, field, hydrothermal vent, black smokers, intertidal zone, cold seeps, bore hole, phylloplane, litteral zone, photic zone, neritic zone, abyssal plane, aphotic zone, rock dwelling, agricultural land/field, athalassic, thalassic, glacier, Indoor or outdoor machine surfaces, dish washing machine surface, house floors, house windows, room).
   2. **Annotation tags:** Site
   3. **Examples:**
      1. “Astronauts were asked to swab 15 surfaces on the International Space Station. Below are their verbatim instructions.” ([PMC5827671](https://europepmc.org/article/MED/29492330)).
      2. “Řimov Reservoir: Representative water samples of epilimnion (0.5 m) and hypolimnion (30 m) were taken on April 20, 2016, from this mesoeutrophic reservoir (South Bohemia, Czech Republic). The sampling site is located at the deepest part (43 m) of the reservoir 250 m from the dam. For more detail about the reservoir, see the reference.” ([PMC6169038](https://europepmc.org/article/MED/30285851))
9. **State**
   1. **Terms to be annotated:** Any term that represents the state or condition of the host or environment from which the sample was collected (e.g. healthy, disease, athletic, obese, lean, age, ethnicity, gender, temperature, salinity, acidity, hypersaline, lentic, marine, geologic, terrestrial, neutral, aquatic, alkaline, brackish, acidic, salty, diffuse flow, oil-contaminated, salt crystalizer, tropical, boreal, hot, meromictic, subglacial, thermal, near-boiling, non-marine, aphotic, polluted).
   2. **Annotation tags:** State
   3. **Examples:**
      1. “We enrolled patients with diagnosis of Anorexia nervosa (AN) referred to the Eating Disorder Unit of ASST Santi Paolo e Carlo in Milan from January 2016 to June 2016.” ([PMC5479564](https://europepmc.org/article/MED/28636668))
      2. “Řimov Reservoir: Representative water samples of epilimnion (0.5 m) and hypolimnion (30 m) were taken on April 20, 2016, from this mesoeutrophic reservoir (South Bohemia, Czech Republic). The sampling site is located at the deepest part (43 m) of the reservoir 250 m from the dam. For more detail about the reservoir, see the reference.” ([PMC6169038](https://europepmc.org/article/MED/30285851))
      3. “Soil from Mad Boon is previously uncharacterized, with dense mangrove forest in one side and hypersaline soil in another side of backwater located in Southeast coast of Tamil Nadu, India.” ([PMC6329363](https://europepmc.org/article/MED/30656204))
10. **Treatment**
    1. **Terms to be annotated:** Any term that represents any treatment applied to the host or environment from which the sample was collected. This can include dietary treatments, medical interventions and therapeutic approaches, drug therapy, infectious challenges or any physical or chemical treatments.
    2. **Annotation tags:** Treatment
    3. **Examples:**
       1. “Infants reported to have never been introduced to formula and to be breastfed were considered to be exclusively breastfed, while those who were ever breastfed and ever formula-fed were considered to be fed with a combination of breast milk and formula regardless of whether or not the infant was still breastfed at the time of stool collection.” ([PMC6033232](https://europepmc.org/article/MED/29973274)).
       2. “Treatment began immediately after weaning (three weeks old). Following the third ABX treatment (six weeks old), mice were administered HDM for five weeks according to the model described earlier.” ([PMC6258300](https://europepmc.org/article/pmc/6258300))
11. **Kit**
    1. **Terms to be annotated:** Any term that represents the name of nucleic acid extraction kit.
    2. **Annotation tags:** Kit
    3. **Examples:**
       1. DNA was extracted with the DNeasy Tissue Extraction Kit (Qiagen) as directed by the manufacturer. ([PMC3250512](https://europepmc.org/article/MED/22235194))
       2. DNA from total gut samples was extracted using the MoBio PowerSoil extraction kit. ([PMC6264799](https://europepmc.org/article/MED/30374168))
       3. DNA was extracted using a QIAamp DNA Stool Mini Kit (Qiagen) according to the manufacturer's instructions and the purified DNA was stored at −20°C until use ([PMC4199679](https://europepmc.org/article/MED/25329397))
12. **Primer**
    1. **Terms to be annotated:** Any term that represents the name of PCR primer used in amplifying the target gene(s).
    2. **Annotation tags:** Primer
    3. **Examples:**
       1. The primers were (341F: 5′-CCTAYGGGRBGCASCAG-3′, 806R: 5′-GGACTACNNGGGTATCTAAT-3′). ([PMC6547527](https://europepmc.org/article/MED/31159860))
       2. The primers were (RP841F: 5′-GACTAGGGATTGGARTGG-3′, Reg1320R: 5′-AATTGCAAAGATCTATCCC-3′). ([PMC6547527](https://europepmc.org/article/MED/31159860))
       3. To estimate total bacterial abundance, quantitative PCR (qPCR) was performed on fecal DNA using the following primers: forward, 5’-ACTCCTACGGGAGGCAGCAGT-3’, reverse, 5’-GTATTACCGCGGCTGCTGGCAC-3’ ([PMC6264799](https://europepmc.org/article/MED/30374168))
13. **Gene**
    1. **Terms to be annotated:** Any terms the represent targeted genes (phylogenetic marker genes) or hypervariable gene regions and subfragments (e.g. 16S rRNA, 18S rRNA, nif, amoA, rpo, V4)
    2. **Annotation tags:** Gene
    3. **Examples:**
       1. Two different PCRs were set up separately with a set of barcoded primers targeting the V3V1 region and V3V5 regions; this analysis examined V3V5 regions.The V3V5 regions of the 16S rRNA[1] gene were amplified by PCR using bar-coded universal primers 354F and 926R (V3-V5) containing the A and B sequencing adaptors (454 Life Sciences, Branford, CT) obtained from Invitrogen. ([PMC3374618](https://europepmc.org/article/MED/22719832))
       2. The DNA was used to amplify the V3-V4 16S rRNA gene variable regions using the primers S-D-Bact-0341-b-S-17 and S-D-Bact-0785-a-A-21. ([PMC6200919](https://europepmc.org/article/MED/30405581))
14. **LS**
    1. **Terms to be annotated:** Any term that represents DNA library source or strategy (e.g. Amplicon, Whole genome shotgun (WGS)/Metagenomic, RNA-Seq, Whole genome amplification sequencing(WGA).
    2. **Annotation tags:** LS
    3. **Examples:**
       1. “Amplicon libraries for the V4-V6 region of 16S rRNA bacterial genes were prepared using fused primers and sequenced using Roche Titanium technology as previously described (82).” ([PMC5080406](https://europepmc.org/article/MED/27822558))
       2. “DNA samples obtained from the study of Gülay et al. (2016) were subjected to shotgun metagenomic sequencing.” ([PMC5113852](https://europepmc.org/article/MED/27128989))
       3. RNA-seq libraries were prepared with the TruSeq RNA sample prep kit by following the manufacturer’s instructions (Illumina, San Diego, CA).” ([PMC4809917](https://europepmc.org/article/MED/26818672))
       4. “Whole genome amplification (WGA) was applied in triplicate for each sample using GenomiPhi V2 (GE Healthcare) following manufacturers’ instructions and the triplicates were pooled.” ([PMC4701944](https://europepmc.org/article/MED/26779149))
15. **LCM**
    1. **Terms to be annotated:** Any term that represents DNA library construction method or layout (e.g. single-end: reading DNA strands from one end, paired-end: reading DNA strands from both ends using reverse and forward primers).
    2. **Annotation tags:** LCM
    3. **Examples:**
       1. We constructed a paired-end library with insert size of ∼350 base pairs for every sample. ([23023125](https://europepmc.org/article/MED/23023125))
       2. The remaining four clones (1A, 3B, 6A and 10A) were sequenced at UNC (Chapel Hill, NC) using an Illumina Hiseq2000 Single-end 50 bp, and library construction was performed according to the manufacturer’s instructions. ([PMC3935994](https://europepmc.org/article/MED/24587220))
16. **Sequencing**
    1. **Terms to be annotated:** Any term that represents the name of the sequencing method or platform
    2. **Annotation tags:** Sequencing
    3. **Examples:**
       1. Nucleotide databases were constructed using either 454 FLX Titanium or Illumina MiSeq sequencing reads from specimens from the above studies. ([PMC4807089](https://europepmc.org/article/MED/27010970))
       2. “Sequencing was performed using the GS Junior Titanium emPCR Kit (Lib-L), the GS Junior Titanium Sequencing Kit, PicoTiterPlate Kit and GS Junior System according to the manufacturer's instructions (Roche 454 Life Sciences, Branford, CT, USA).“([PMC4444838](https://europepmc.org/article/MED/26074890))
