# Supplementary material for: A machine learning framework for discovery and enrichment of metagenomics metadata from open access publications
Source: Gigascience. 2022 Aug 11;11:giac077. doi: 10.1093/gigascience/giac077 (PMC9366992; doi:10.1093/gigascience/giac077)
Supplement: giac077_Response_to_Reviewer_Comments_Original_Submission [file giac077_response_to_reviewer_comments_original_submission.pdf]

Author's Response To Reviewer Comments

Close

Reviewer #1:
Figure 4 compares the annotations deposited by authors in the ENA to the authors' predictions from EPMC. This is a very useful comparison, and it would also be helpful for users of the published annotations, especially when there are non-identical or unique annotations. Are ENA annotations shown in addition to predicted annotations for users of the tools? I did see this feature mentioned in MGnify in the final Discussion paragraph, but I wasn't clear about within EPMC.

• The metadata terms found in EPMC are solely from the terms found within the article. While we appreciate that having additional metadata terms that a researcher may have submitted within the sequence record, there would not be a natural way of displaying this in EPMC, especially if the term was not found in EPMC. Similarly, ENA will not display metadata terms identified EPMC, as the records are owned by the submitter and cannot be modified. Thus, this functionality is performed by MGnify, but only on a study-by-study basis. As highlighted by the reviewer this comparison is useful but is restricted to this paper. Conceivably, it would be possible to recreate the analysis by using the respective resource APIs. We have tried to make this clearer in the manuscript.

Table 4: In the MIxS column it appears that the first term given is from MIxS, and the second term in parentheses is the authors' annotation term, but this isn't clear from the table or caption.

• MIxS (entity type) has been added to the MIxS column header.

The sentence "On the other hand, training 16 individual NER models (one model per entity) on all curated sentences provided extensive negative dataset and considerable advantage for training overlapping entities, …" is unclear, please clarify.

• We have tried to make this sentence clearer. More specifically, this sentence states that curating individual NER models addressed the overlapping entities issue. Furthermore, training each entity on all curated sentences, as mentioned in the methods, provided rich negative contexts. We deleted the negative context part to avoid confusion.

The pipeline continuously providing metagenomics annotations for new EPMC publications, and the user tools for accessing them, are mentioned almost only in passing in the Results. Many readers might miss this and think that all of this work was an interesting exercise but with results they can't readily use. Since the user tools built into EPMC, SciLite, and an API will be the part of greatest interest to many microbiome researchers, I recommend highlighting the availability of user tools in the Abstract, Introduction (which currently in my opinion has more than necessary on ML methods and is too brief on the final paragraph of how this work fills the previous gap), and Discussion (the mention in the Discussion that "This framework has been integrated into Europe PMC and further propagated to MGnify" does not make clear what this means to users).

• We appreciate this suggestion and have added additional sentences to the abstract and introduction to underscore how the algorithm/pipeline is used within EPMC and MGnify services and will be updated on a regular basis.

Reviewer #2:

Possibly a few sentences could be added to the discussion regarding application of the software beyond metagenomics (for example could it be applied to more broad annotation databases such as those of

newly sequenced genomes).

• This is an interesting suggestion/comment. The reviewer is correct that there are many metadata terms that are identified by our models that would be equally applicable to other databases, such as nucleotide sequence records (where there will be the biggest overlap due to the use of the MIxS terms) that use other ‘omics techniques in sequencing biosamples beyond metagenomics. Conceivably, using the different biological resource accession numbers detected in EPMC, these models could be more widely applied to other articles and subsequently consumed by the source database providing the accession number. We have included some text at the end of the discussion to reflect this, but this would be beyond the scope of this article.

Also a comment whether in the opinion of the authors other approaches for machine learning such as artificial neural networks / deep learning would yield different results than the approach used.

• We have included a brief description of other state-of-the-art models in the discussion section. These models were pretrained on wikipedia articles and have outperformed BERT or showed comparable performance in text classification tasks. However, they do not perform well on NER tasks. Fine-tuning these models with the metagenomics dataset is not expected to show better performance unless they were pretrained on a much larger training set than the one that was available to us (e.g., millions of PMC papers) as BioBERT and addressed the unknown token issue we have addressed in tensorflow BERT script. This is addressed in the discussion, where we have added further clarification. Also, it is worth noting that we have attempted to fine-tune BERT, XLNET, RoBERTa and ELECTRA models (pretrained on wikipedia) with metagenomics training dataset to explore if other model architectures will show better performance. And, initial performance metrics revealed BERT outperforming the other models. However, this was a preliminary fine-tuning attempt with wikipedia-pretrained models and an accurate model’s comparison will require pretraining similar architecture models on millions of biomedical publications to address the complexity of novel metagenomics concepts (unknown tokens)

Close
