# Supplementary material for: A machine learning framework for discovery and enrichment of metagenomics metadata from open access publications
Source: Gigascience. 2022 Aug 11;11:giac077. doi: 10.1093/gigascience/giac077 (PMC9366992; doi:10.1093/gigascience/giac077)
Supplement: giac077_Response_to_Reviewer_Comments_Revision_1 [file giac077_response_to_reviewer_comments_revision_1.pdf]

Author's Response To Reviewer Comments

Close

Dear Dr Nogoy,

Re: Manuscript submission: “A machine learning framework for discovery and enrichment of metagenomics metadata from open access publications''.

Thank you for your consideration of our manuscript entitled ‘A machine learning framework for discovery and enrichment of metagenomics metadata from open access publications’. We are pleased that our manuscript is acceptable for publication. As requested, we have addressed your comments in the manuscript and added “Availability of Supporting Data” section, cited GigaScience database under this section, included GigaDB DOI in the references, registered EMERALD metagenomics annotations pipeline in BioTools and cited biotools identifier in the manuscript under “Availability of Source Code”.

We hope the manusctipt can now progress to the next step.

Yours sincerely

Johanna McEntyre
Associate Director of Services

Close
