# Supplementary material for: A machine learning framework for discovery and enrichment of metagenomics metadata from open access publications
Source: Gigascience. 2022 Aug 11;11:giac077. doi: 10.1093/gigascience/giac077 (PMC9366992; doi:10.1093/gigascience/giac077)
Supplement: giac077_Reviewer_1_Report_Original_Submission — Levi D Waldron -- 4/12/2022 Reviewed [file giac077_reviewer_1_report_original_submission.pdf]

Reviewer Report

Title: A machine learning framework for discovery and enrichment of metagenomics metadata from open access publications

Version: Original Submission **Date:** 4/12/2022

Reviewer name: Levi D Waldron

Reviewer Comments to Author:

This manuscript describes a machine learning framework for extracting study-level metadata for metagenomics studies from literature in the Europe PMC. Named Entity Recognition (NER) models were developed individually for each 16 metadata features involving environment, laboratory methods, setting, etc. An interesting approach was used involving creating "teacher" models from labeled datasets, then developing "student" models from predictions of the "teacher" models on a larger training dataset. Validation of "student" models was assessed in labeled datasets not included in the original training, demonstrating generally quite high accuracy, with the lower F1 statistics being cases where manual curation identified additional terms, resulting in high precision but lower recall. Overall, the accuracy is certainly high enough to be useful. The pipeline is applied to more than 100K studies from Europe PMC, including ~20K describing metagenomics studies. This open-source pipeline is stated to be now continually updating new submissions to Europe PMC, and its results now being available to researchers at large through several options: 1) search through the Europe PMC website, 2) a SciLite application, and 3) the "annotations" API. As a result this is not only a method being proposed, but a metadata resource along with tools of practical significance for most microbiome researchers. The methods and the resources produced are of high impact and already of high quality, and I have only an enhancement suggestion and some manuscript-related comments.
Enhancement suggestion:
Figure 4 compares the annotations deposited by authors in the ENA to the authors' predictions from EPMC. This is a very useful comparison, and it would also be helpful for users of the published annotations, especially when there are non-identical or unique annotations. Are ENA annotations shown in addition to predicted annotations for users of the tools? I did see this feature mentioned in MGnify in the final Discussion paragraph but I wasn't clear about within EPMC.
Minor:
Table 4: In the MIxS column it appears that the first term given is from MIxS, and the second term in parentheses is the authors' annotation term, but this isn't clear from the table or caption.
The sentence "On the other hand, training 16 individual NER models (one model per entity) on all curated sentences provided extensive negative dataset and considerable advantage for training overlapping entities, …" is unclear, please clarify.
The pipeline continuously providing metagenomics annotations for new EPMC publications, and the user tools for accessing them, are mentioned almost only in passing in the Results. Many readers might miss this and think that all of this work was an interesting exercise but with results they can't readily use. Since the user tools built into EPMC, SciLite, and an API will be the part of greatest interest to many

microbiome researchers, I recommend highlighting the availability of user tools in the Abstract, Introduction (which currently in my opinion has more than necessary on ML methods and is too brief on the final paragraph of how this work fills the previous gap), and Discussion (the mention in the Discussion that "This framework has been integrated into Europe PMC and further propagated to MGnify" does not make clear what this means to users).

Methods

Are the methods appropriate to the aims of the study, are they well described, and are necessary controls included? Choose an item.

Conclusions

Are the conclusions adequately supported by the data shown? Choose an item.

Reporting Standards

Does the manuscript adhere to the journal's guidelines on minimum standards of reporting? Choose an item.

Choose an item.

Statistics

Are you able to assess all statistics in the manuscript, including the appropriateness of statistical tests used? Choose an item.

Quality of Written English

Please indicate the quality of language in the manuscript: Choose an item.

Declaration of Competing Interests

Please complete a declaration of competing interests, considering the following questions:

- Have you in the past five years received reimbursements, fees, funding, or salary from an organisation that may in any way gain or lose financially from the publication of this manuscript, either now or in the future?
- Do you hold any stocks or shares in an organisation that may in any way gain or lose financially from the publication of this manuscript, either now or in the future?
- Do you hold or are you currently applying for any patents relating to the content of the manuscript?
- Have you received reimbursements, fees, funding, or salary from an organization that holds or has applied for patents relating to the content of the manuscript?

- Do you have any other financial competing interests?
- Do you have any non-financial competing interests in relation to this paper?

If you can answer no to all of the above, write 'I declare that I have no competing interests' below. If your reply is yes to any, please give details below.

I declare that I have no competing interests.

I agree to the open peer review policy of the journal. I understand that my name will be included on my report to the authors and, if the manuscript is accepted for publication, my named report including any attachments I upload will be posted on the website along with the authors' responses. I agree for my report to be made available under an Open Access Creative Commons CC-BY license (http://creativecommons.org/licenses/by/4.0/). I understand that any comments which I do not wish to be included in my named report can be included as confidential comments to the editors, which will not be published.

Choose an item.

To further support our reviewers, we have joined with Publons, where you can gain additional credit to further highlight your hard work (see: https://publons.com/journal/530/gigascience). On publication of this paper, your review will be automatically added to Publons, you can then choose whether or not to claim your Publons credit. I understand this statement.

Yes Choose an item.
